# The Affinity of Elongated Membrane-Tethered Ligands Determines Potency of T Cell Receptor Triggering

**DOI:** 10.3389/fimmu.2017.00793

**Published:** 2017-07-10

**Authors:** Bing-Mae Chen, Mohammad Ameen Al-Aghbar, Chien-Hsin Lee, Tien-Ching Chang, Yu-Cheng Su, Ya-Chen Li, Shih-En Chang, Chin-Chuan Chen, Tsai-Hua Chung, Yuan-Chun Liao, Chau-Hwang Lee, Steve R. Roffler

**Affiliations:** ^1^Institute of Biomedical Sciences, Academia Sinica, Taipei, Taiwan; ^2^Taiwan International Graduate Program in Molecular Medicine, National Yang-Ming University and Academia Sinica, Taipei, Taiwan; ^3^Institute of Biochemistry and Molecular Biology, National Yang-Ming University, Taipei, Taiwan; ^4^Research Center for Applied Sciences, Academia Sinica, Taipei, Taiwan; ^5^Institute of Biophotonics, National Yang-Ming University, Taipei, Taiwan; ^6^Department of Physics, National Taiwan University, Taipei, Taiwan; ^7^Graduate Institute of Medicine, College of Medicine, Kaohsiung Medical University, Kaohsiung, Taiwan

**Keywords:** affinity, T cell receptor triggering, artificial antigen-presenting cell, pMHC, anti-CD3 scFv, OKT3, BC3, 2C11

## Abstract

T lymphocytes are important mediators of adoptive immunity but the mechanism of T cell receptor (TCR) triggering remains uncertain. The interspatial distance between engaged T cells and antigen-presenting cells (APCs) is believed to be important for topological rearrangement of membrane tyrosine phosphatases and initiation of TCR signaling. We investigated the relationship between ligand topology and affinity by generating a series of artificial APCs that express membrane-tethered anti-CD3 scFv with different affinities (OKT3, BC3, and 2C11) in addition to recombinant class I and II pMHC molecules. The dimensions of membrane-tethered anti-CD3 and pMHC molecules were progressively increased by insertion of different extracellular domains. In agreement with previous studies, elongation of pMHC molecules or low-affinity anti-CD3 scFv caused progressive loss of T cell activation. However, elongation of high-affinity ligands (BC3 and OKT3 scFv) did not abolish TCR phosphorylation and T cell activation. Mutation of key amino acids in OKT3 to reduce binding affinity to CD3 resulted in restoration of topological dependence on T cell activation. Our results show that high-affinity TCR ligands can effectively induce TCR triggering even at large interspatial distances between T cells and APCs.

## Introduction

T cells play a central role in the adaptive immune response by killing viral-infected or cancer cells and by regulating other immune cells. T cell activation is initiated by binding of a cognate T cell receptor (TCR) to short stimulatory peptides in the context of major histocompatibility complex (pMHC) (1, 2). The TCR α/β chains have short cytoplasmic tails, but all proximal signaling events are transduced through the associated CD3 molecules (3). The individual CD3 molecule possesses one to three intracellular motifs for tyrosine phosphorylation, known as immune-receptor tyrosine-based activation motifs (ITAMs) (3, 4). Engagement of a specific TCR by a pMHC triggers the phosphorylation of CD3 ITAMs by Src-family kinases Lck and Fyn, which promotes binding and phosphorylation of the Src homology 2 (SH2)-containing protein, ZAP70. Consequently, ZAP70 phosphorylates the adapter proteins, linker for T cell activation (LAT), and SH2 domain-containing leukocyte protein of 76 kDa (SLP76), which leads to activation of downstream signaling and expression of effector functions (5, 6).

T cell receptor triggering is still not completely understood. In general, TCR clustering, and relative movement of key receptors and ligands on the plasma membrane of T cells and antigen-presenting cells (APCs) are believed to play crucial roles in the initiation of TCR signaling [reviewed in Ref. (7)]. Many models have been proposed to explain how binding to pMHC molecules on APCs can initiate TCR triggering including increased phosphorylation of CD3 ITAMs by induced proximity of Lck associated with CD4 or CD8 co-receptors (8), reorganization of kinase-rich raft microdomains near the TCR complex by CD28 costimulation (9), and conformational changes in the CD3 cytoplasmic tail upon pMHC/TCR ligation (10).

Kinetic segregation (KS) is a popular model that posits that the small dimensions of the TCR and pMHC complex physically exclude inhibitory transmembrane receptor protein tyrosine phosphatases such as CD45 and CD148 due to their large ectodomains, allowing sustained phosphorylation of CD3 chains by Lck and Fyn (11). Direct evidence for the KS model is provided by studies showing that increasing the dimensions of pMHC and TCR ligand molecules results in progressive loss of their ability to activate T cells, supposedly due to reduced segregation of inhibitory receptor tyrosine phosphatases from engaged TCRs (11–14).

**Figure 1:**
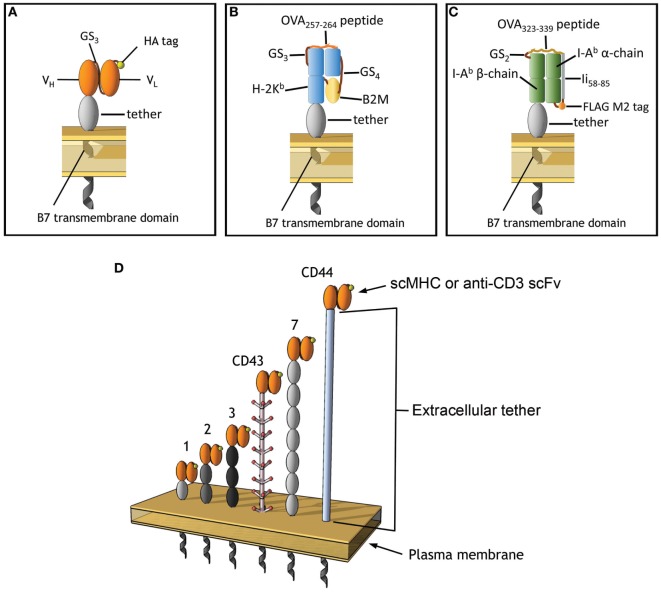
Membrane-tethered T cell receptor ligands. **(A)** Anti-CD3 scFv include an N-terminal HA epitope tag, the variable light, and heavy chains are connected with 3 repeats of GS linker (G_4_S). **(B)** A single-chain MHC (scMHC) I molecule that presents OVA_257–264_ in the context of H-2K^b^, 4 and 3 repeats of GS linkers were used between the B2M and after the OVA peptide, respectively. **(C)** A scMHC II molecule that presents OVA_323–339_ in the context of I-A^b^. **(D)** The scFv and scMHC molecules were anchored on the membrane of 3T3 fibroblasts *via* protein tethers with different dimensions. Ovals represent Ig-like domains. The tethers are derived from BGP-1 (one Ig domain), a monomeric IgG_1_ Fc domain (two Ig domains), CD66 (three Ig domains), CD43 (extended rod-like structure), PTK-7 (seven Ig domains), or CD44 (unknown structure).

In the present study, we further investigated the interaction of ligand affinity and receptor dimensions in TCR triggering by creating artificial APCs (aAPCs) of 3T3 cells that express membrane-tethered anti-CD3 single-chain variable fragment (scFv) with different dimensions and affinities or class I and II single-chain MHC (scMHC) and examining their ability to elicit T cell responses (Figure 1). We found that elongating the dimensions of low-affinity ligands results in progressive loss of T cell activation similar to what is predicted by the KS model. However, high-affinity ligands can effectively trigger T cell activation regardless of their topology, suggesting that the KS model is insufficient to explain T cell activation.

## Materials and Methods

### Cell Lines and Animals

OKT3 hybridoma cells were obtained from the Bioresource Collection and Research Center (Hsinchu, Taiwan). BALB/c 3T3 cells, HT29 human colon cancer cells, BC3 hybridoma cells, HB65 hybridoma cells, and Jurkat human T cells were from the American Type Culture Collection (Manassas, VA, USA). 2B4 mouse T cells were kindly provided by Dr. Ming-Zong Lai, Institute of Molecular Biology, Academia Sinica. The B3Z mouse T cell hybridoma was kindly provided by Dr. Ya-Wun Yang, School of Pharmacy, College of Medicine, National Taiwan University. OT-I (15) and OT-II (16) transgenic mice were a kind gift from Dr. Nan-Shih Liao, Institute of Molecular Biology, Academia Sinica. Mouse splenic T cells were obtained from BALB/c, C57BL/6, OT-I, or OT-II mice *via* purification over nylon wool.

Human whole blood, obtained from healthy donors by the Taipei City Blood Bank mentioned already in ethics statement.

### Antibodies and Reagents

Rat anti-HA (clone 3F10) was purchased from Roche (Mannheim, Germany). The 25D-1.16 antibody, which recognizes K^b^-SINFEKL complexes, was generously provided by Dr. Ron Germain, National Institutes of Health (Bethesda, MD). Mouse anti-HA (clone 16B12) was from Covance (Berkeley, CA, USA). FITC-labeled anti-CD8, PE-labeled CD44, Alexa Fluor 647-labeled anti-CD62L, PE-labeled AF6-88.5 (anti-H-2K^b^), and FITC-labeled AF6-120.1 (anti-I-A^b^) antibodies were from BD Biosciences (East Rutherford, NJ, USA). Rat anti-CD66acd antibody was from AbD Serotec (Kidlington, UK). Rabbit anti-6xHis Tag antibody was from Bioman Scientific (Jhonghe, Taiwan). HRP-conjugated affinipure donkey anti-mouse IgG, HRP-conjugated affinipure goat anti-rabbit IgG, HRP-conjugated affinipure goat anti-rat IgG, HRP-conjugated streptavidin, goat anti-mouse Ig(A + G + M), and goat anti-human Ig(A + G + M) were from Jackson ImmunoResearch (West Grove, PA, USA). FITC-conjugated goat F(ab′)_2_ anti-mouse IgG Fc was from ICN Pharmaceuticals (Aurora, OH, USA). Mouse anti-β-actin (Clone AC-74) was from Sigma-Aldrich (St. Louis, MO, USA). Biotin-conjugated mouse anti-phosphotyrosine (clone 4G10) and rabbit polyclonal anti-ZAP-70 were from Millipore (Temecula, CA, USA). 2C11, BC3, and OKT3 antibodies were purified from ascites produced in BALB/c mice by affinity chromatography using Protein G Sepharose (GE Healthcare Bio-Sciences AB, Uppsala, Sweden).

### Recombinant DNA

We used the murine B7.1 transmembrane and cytoplasmic domains to express and tether ligands on the surface of 3T3 APCs. The construction of p2C11-B7, p2C11-BGP-B7 (2C11-1), p2C11-γ1-B7 (2C11-2d), p2C11-CD44-B7 (2C11-CD44), and p2C11-CD43-B7 (2C11-CD43) have been described (14, 17, 18). We used PCR to amplify the CH2-CH3 domains from human IgG1 excluding the hinge region using p2C11-γ1-B7 as a template, the modified γ1 (mγ1) was used to replace BGP in p2C11-BGP-B7 to generate p2C11-mγ1-B7 (2C11-2). DNA fragments encompassing the ectodomains of human CD66 and PTK-7 with flanking *Sal I* sites were amplified from HT29 cells by RT-PCR. These DNA fragments were inserted in place of the BGP fragment in p2C11-BGP-B7 to generate p2C11-CD66-B7 (2C11-3) and p2C11-PTK-B7 (2C11-7), respectively. The γ1 fragment in pLNCX-phOx-γ1-B7 (18) was replaced with the BGP fragment in p2C11-BGP-B7 to generate the control scFv construct pLNCX-phOx-BGP-B7 (phOx-1).

The constructs encoding for BC3 and OKT3 were prepared as described by Chou et al. (19). Briefly, the variable light (V_L_) and heavy (V_H_) chain cDNA sequences of BC3 and OKT3 antibodies were amplified by RT-PCR from RNA isolated from BC3 and OKT3 hybridoma cells. The gene fragments were digested with *SfiI* and *SalI* restriction enzymes and replaced 2C11 in p2C11-B7 to generate the pBC3-B7 and pOKT3-B7, respectively. The genes coding spacers (BGP, mγ1, CD66, PTK, CD43, and CD44) were inserted into the unique *SalI* restriction sites in pBC3-B7 and pOKT3-B7 to generate vectors coding for BC3-1, BC3-2, BC3-3, BC3-7, BC3-CD43, BC3-CD44 and OKT3-1, OKT3-2, OKT3-3, OKT3-7, OKT-CD43, and OKT3-CD44, respectively. All transgenes were cloned into the pLNCX retroviral vector (Clontech, CA) to yield recombinant retroviral particles for generation of stable 3T3 cell lines.

To produce soluble scFv, the transmembrane anchor sequence in p2C11-B7 was replaced with a polyhistidine tag (6xHis). BC3 and OKT3 scFv sequences were digested by *SfiI* and *SalI* and replaced 2C11 in p2C11-6xHis.

Class I scMHC was constructed as previously described by Yu et al. (20). The construct encoded the signal peptide sequence of beta-2-microglobulin (β2m), the SIINFEKL (OVA_257–264_) antigen, a 15 amino acid linker (G_4_S)_3_, mature β_2_m, a 20 amino acid linker (G_4_S)_4_, and the extracellular portion of mature H-2K^b^ (amino acids 22–296). The cDNA for the signal peptide, SIINFEKL, first linker, and the first 10 amino acids of the β_2_m gene, flanked by *HindIII* and *BstZ17I* restriction sites, were generated by assembly PCR. A fragment of the mature β2m gene (amino acid 9 to the stop codon) and the second flexible linker, flanked by *BstZ171* and *BspEI* restriction sites, was amplified from C57BL/6 splenocyte RNA by RT-PCR. The extracellular portion of the mature form of the H-2K^b^ gene (amino acids 22–296) with flanking *BspEI* and *SalI* restriction sites was likewise amplified from C57BL/6 splenocyte RNA by RT-PCR. A negative control scMHC molecule (cK^b^) was constructed with the human papillomavirus peptide DRAHYNIV (E7_48–55_). The DNA fragments were assembled and inserted in place of the 2C11 scFv gene in pLNCX-2C11-B7, pLNCX-2C11-BGP-B7, pLNCX-2C11-CD66-B7, and pLNCX-2C11-CD44-B7 to create the corresponding class I scMHC (ovK^b^) retroviral vectors.

A MHC class II single-chain trimer (SCT) was constructed as previously described by Thayer et al. (21). The I-A^b^ α-chain extracellular domain, including the signal peptide, was fused to a 10 amino acid (G_4_S)_2_ linker, a fragment of the mouse invariant chain (residues 58–85), followed by the ova-derived antigen (residues 323–339), ISQAVHAAHAEINEAGR, a 19 amino acid linker containing the Flag-M2 tag (GGGSGDYKDDDDKGGGGGS), the I-A^b^ β-chain extracellular domain, and the original transmembrane and cytoplasmic domain. The extracellular portion of the I-A^b^ α-chain from amino acids 1–219 with flanking *HindIII* and *AgeI* restriction sites was amplified by RT-PCR from C57BL/6 mice splenocyte RNA. DNA for the first linker, invariant chain from amino acids 58–85, the OVA_323–339_ peptide, the second linker, and the first 7 amino acids of the mature form of the β-chain (amino acids 20–26), flanked by *AgeI* and *BstZ171* restriction enzyme sites, was generated by synthetic PCR. cDNA for the extracellular portion of the mature β-chain from amino acids 26–209, flanked by *BstZ171* and *SalI* restriction sites, was amplified by RT-PCR from C57BL/6 mice splenocyte RNA. The assembled class II SCT gene (ovA^b^) was inserted in place of the 2C11 scFv gene in pLNCX-2C11-B7, pLNCX-2C11-BGP-B7, pLNCX-2C11-CD66-B7, pLNCX-2C11-PTK-B7, and pLNCX-2C11-CD44-B7 to create the corresponding class II SCT (ovA^b^) retroviral vectors.

### Generation of Stable Cell Lines

Permanent 3T3 APCs cell lines expressing tethered MHC, tethered, or soluble scFv were generated by retroviral infection as described (12, 22). After selection in antibiotic-containing culture medium, stable transfectants were sorted on FACSAria IIu cell sorter for similar surface expression of the tethered ligands.

### Purification of Mouse and Human T Lymphocytes

Human peripheral mononuclear cells (PBMCs) were obtained *via* purification of healthy human whole blood (Taipei Blood Bank, Taiwan) by panning on a culture dish for 30 min at 37°C. Unbound cells were collected and incubated in culture dish precoated with goat anti-human Ig(A + G + M) for 30 min to remove the B cells. Mouse splenic T cells were purified from minced mouse spleens. RBCs were lysed by ACK lysis buffer (Gibco BRL, CA, USA). After panning on a culture dish for 30 min at 37°C, unbound cells were incubated in culture dish precoated with goat anti-mouse Ig(A + G + M) as described above. Human or mouse T cells were applied to a nylon wool-packed column (Polyscience, Inc.) equilibrated with serum-containing medium for further purification. After incubation for 1 h, purified T cells were eluted for further assays. OT-I CD8^+^ T cells were sorted by FACS as CD8^+^, CD44^lo^, CD62L^hi^ (naive), CD8^+^, CD44^hi^, CD62L^hi^ (central memory), and CD8^+^, CD44^hi^, CD62L^lo^ (effector memory) T cell populations.

### Flow Cytometer Analysis

Purified mouse or human T cells, Jurkat T cells, 2B4 T cells, or 3T3 APCs were stained with commercial antibodies at the recommended dilutions. The dead cells were gated out by staining with 5 µg/ml Propidium Iodide (Sigma). Mean fluorescence intensities (MFIs) were analyzed with FlowJo (Tree Star, San Carlos, CA, USA).

### Transmission Electron Microscopy

5 × 10^5^ 3T3/OKT3-1, OKT3-2, OKT3-3, OKT3-CD43, OKT3-7, or OKT3-CD44 cells were grown overnight on an Aclar fluoropolymer film (Electron Microscopy Sciences, Hatfield, PA, USA) in a 24-well plate and incubated with 5 × 10^5^ Jurkat T cells for 2 h at 37°C. After washing with PBS, the cells were fixed in a mixture of 2.5% glutaraldehyde and 4% paraformaldehyde in 0.1 M cacodylate buffer (pH 7.3) for 2 h, and then post-fixed in osmium tetroxide for 1 h. Samples were washed with 0.1 M cacodylate buffer three times (15 min/wash) and then dehydrated in a series of ethanol from 30 to 100%. After dehydration, the samples were placed in acetone and infiltrated with Spurr’s resin. Polymerization of Spurr’s resin was performed by heating to 60°C for 48 h. Ultrathin sections (60–90 nm) were cut on a Reichart-Jung Ultracut E microtome and then mounted on 300 mesh copper grids. The sections were stained in saturated aqueous uranyl acetate and Reynold’s lead citrate, washed with distilled water, and examined on a Hitachi H-7000 transmission electron microscope at 150,000× magnification. Analysis of intercellular distances was performed with Adobe Photoshop CS5 by dividing the conjugation area between two cells into equally spaced sections (11–20) and measuring the distance at each point. Nine to 15 individual cell conjugates were analyzed for each group.

### Soluble scFv ELISA

Microtiter plates were coated for 1 h with 10 µg/mL poly-l-lysine (50 μL/well). The wells were washed once with PBS, and 1 × 10^7^ Jurkat or 4 × 10^7^ 2B4 T cells in 100 µL PBS were added to the wells. The plates were centrifuged at 1,000 × *g* for 5 min and the cells were fixed by addition of 50 µL 4% paraformaldehyde for 30 min at room temperature. The plates were washed with PBS, deactivated for 30 min with 100 mM glycine, and blocked with 5% skim milk in PBS. Soluble OKT3, BC3, and 2C11 scFv were purified from the culture medium of stable 3T3 producer cells using Talon Ni^2+^ affinity chromatography (GE Healthcare). Serial dilutions of purified OKT3 or BC3 scFv were added to plates coated with Jurkat T cells, whereas 2C11 scFv was incubated with plates coated with 2B4 T cells. After 1.5 h, the plates were washed twice with 0.05% Tween-20/PBS and twice with PBS. scFv binding was determined by sequential addition of rabbit anti-6xHis Tag antibody and HRP-conjugated goat anti-rabbit IgG.

### T Cell Binding Assay and Competition Binding Assay

1.5 × 10^6^ Jurkat or 2B4 T cells were labeled with 4 µM calcein-AM (Sigma) for 1.5 h in serum-free culture medium and briefly centrifuged onto 1.5 × 10^5^ 3T3 cells expressing membrane-tethered scFv and allowed to bind for 1 h at 37°C. After washing with PBS containing 0.495 mM MgCl_2_ and 0.9 mM CaCl_2_ six times for 3 min each time, bound cells were trypsinized, transferred to a black microtiter fluorescence plate, and fluorescence was measured at 485/525 nm (excitation/emission) on a SpectraMax Gemini EM fluorescence microplate reader (Molecular Device, CA, USA). For the binding competition assay, defined concentration of congenic soluble anti-CD3 whole IgG antibodies or 10 µg/ml anti-influenza virus A nucleoprotein control antibody was added with the calcein-AM labeled T cells to 3T3 cell monolayers expressing membrane-tethered scFv. After 1 h incubation at 37°C, unbound cells were removed by thorough washing and the fluorescence of typsinized T cells was determined as above. The competition results were normalized for the different expression levels of each membrane-tethered scFv by dividing the concentration of soluble anti-CD3 antibody added to wells by the MFI of membrane scFv on 3T3 cells as measured by immunofluorescence staining with anti-HA antibody followed by quantification on a flow cytometer.

### Measuring the Surface Density of scFv on APCs

The ligand density on the surface of APCs was measured by using an Fc-specific capture antibody beads standard (Quantum™ Simply Cellular^®^, Bangs Laboratories, Inc.). Briefly, ligands on APCs were stained with saturated amounts of mouse anti-HA IgG, followed by saturated amounts of FITC-labeled anti-mouse IgG. Similarly, the same number of standard beads was stained with the same saturated primary and secondary antibodies. FACS analysis was used to generate a standard curve for the antibody-binding capacity. The surface density was calculated by dividing the antibody-binding capacity values to the surface area of the APCs.

### T Cell Activation

T cell proliferation was measured as described (12). Briefly, defined numbers of APCs were treated with 0.5 mg/ml mitomycin C (MMC) for 1.5 h, washed with PBS three times, and resuspended in RPMI supplemented with 10% FBS. APCs were mixed with purified T cells in triplicate and briefly centrifuged to initiate contact in round-bottom 96-well plates. 30 ng/ml PMA was added to provide costimulation to T cells. After 48 h, 1 μCi ^3^H-thymidine was added for 16 h before measuring radioactivity in a Topcount scintillation counter.

To measure cytokine secretion, graded numbers of MMC-treated APCs were mixed with 5 × 10^4^ Jurkat, 2B4, B3Z, or purified T cells and 30 ng/ml PMA in triplicate in 96-well microplates for 24 or 48 h. Cytokine concentrations were measured by ELISA using BD OptEIA kits for IL-2 and IFN-γ (BD Biosciences).

To assess tyrosine phosphorylation of CD3ζ, Jurkat cells were incubated with APCs expressing scFv for 0, 2, 5, or 10 min at 37°C and immediately lysed in protein lysis buffer (2% Triton X-100, 20 mM 0.5 M Tris at pH 7.6, 300 mM NaCl, 10 mM EDTA, 100 mM NaF, 60 mM Na_4_P_2_O_7_, 2 mM 0.5 M Na_3_VO_4_, and protease inhibitor cocktail from Sigma) for 1 h at 4°C. The mixture was centrifuged at 16,000 × *g* for 30 min and the supernatants were incubated with 15 µl mouse anti-CD3ζ agarose beads (Santa Cruz) for 1 h at 4°C. After washing twice with protein lysis buffer and centrifugation at 16,000 × *g* for 30 min, the beads were boiled in 15 µl of SDS-PAGE buffer and resolved by electrophoresis. For immunoblotting, mouse anti-human CD3ζ antibody (Santa Cruz) and biotin-conjugated mouse anti-phosphotyrosine (Millipore) were used. Detection of tyrosine phosphorylation on ZAP-70 was achieved in a similar fashion by immunoprecipitation with rabbit polyclonal anti-ZAP-70 (Millipore) and immunoblotted with anti-phosphotyrosine and anti-ZAP-70 antibody.

### Generation of Low-Affinity OKT3 scFv

Site-directed mutagenesis was performed using the QuickChange^®^ site-directed mutagenesis kit (Stratagene, La Jolla, CA, USA) to introduce all possible amino acids correspondence to positions R55 and Y57 in the OKT3 V_H_ region gene. These two amino acid positions were selected based on its binding importance as in the crystal structure of OKT3 bound to CD3ε (23, 24). The combinatorial OKT3 scFv library was subcloned into pLNCX-OKT3-1 and a mammalian cell surface expression library was generated by retroviral transduction of 3T3 fibroblasts at a multiplicity of infection of ~0.1. Two weeks after selection of stable cell lines in G418-containing medium, cells that expressed membrane-anchored OKT3 scFv variants as determined by positive immunostaining with PE-conjugated mouse anti-CD66a (BGP-1) (R&D Systems, Minneapolis, MN, USA) were individually arrayed into wells of 96-well culture plates with a FACSVantage DiVa. Confluent monolayers of cells were screened for possible lower binding activity as judged by their ability to bind Jurkat T cells. Twelve clones were further screened by examining the ability of soluble OKT3 to compete calcein-AM labeled Jurkat T cells to 3T3 cells expressing membrane-anchored OKT3 scFv variants as described above. The cDNA sequences of the OKT3 scFv variants (M55/A57) symbolized as (OKT3^MA^) and (L55/T57) symbolized as (OKT3^LT^) were amplified by RT-PCR from RNA isolated from the corresponding 3T3 cells. The generated mutations were determined by sequencing. OKT3^MA^ and OKT3^LT^ cDNAs were inserted into surface display vectors to generate plasmids with the BGP, mγ1, CD43, or CD44 spacers between the scFv and transmembrane domains. Stable 3T3 cells expressing OKT3^MA^ and OKT3^LT^ with various dimensions were generated as described above.

### Statistical Analysis

Statistical analysis was assessed with GraphPad Prism 5 software. Significance of differences between mean values were estimated using the one-way ANOVA (Kruskal–Wallis one-way analysis of variance), or by non-parametric unpaired *t*-test with Welch’s correction. The statistical significance was set at *p* < 0.05.

## Results

### Membrane-Tethered Anti-CD3 scFv

**Figure 2:**
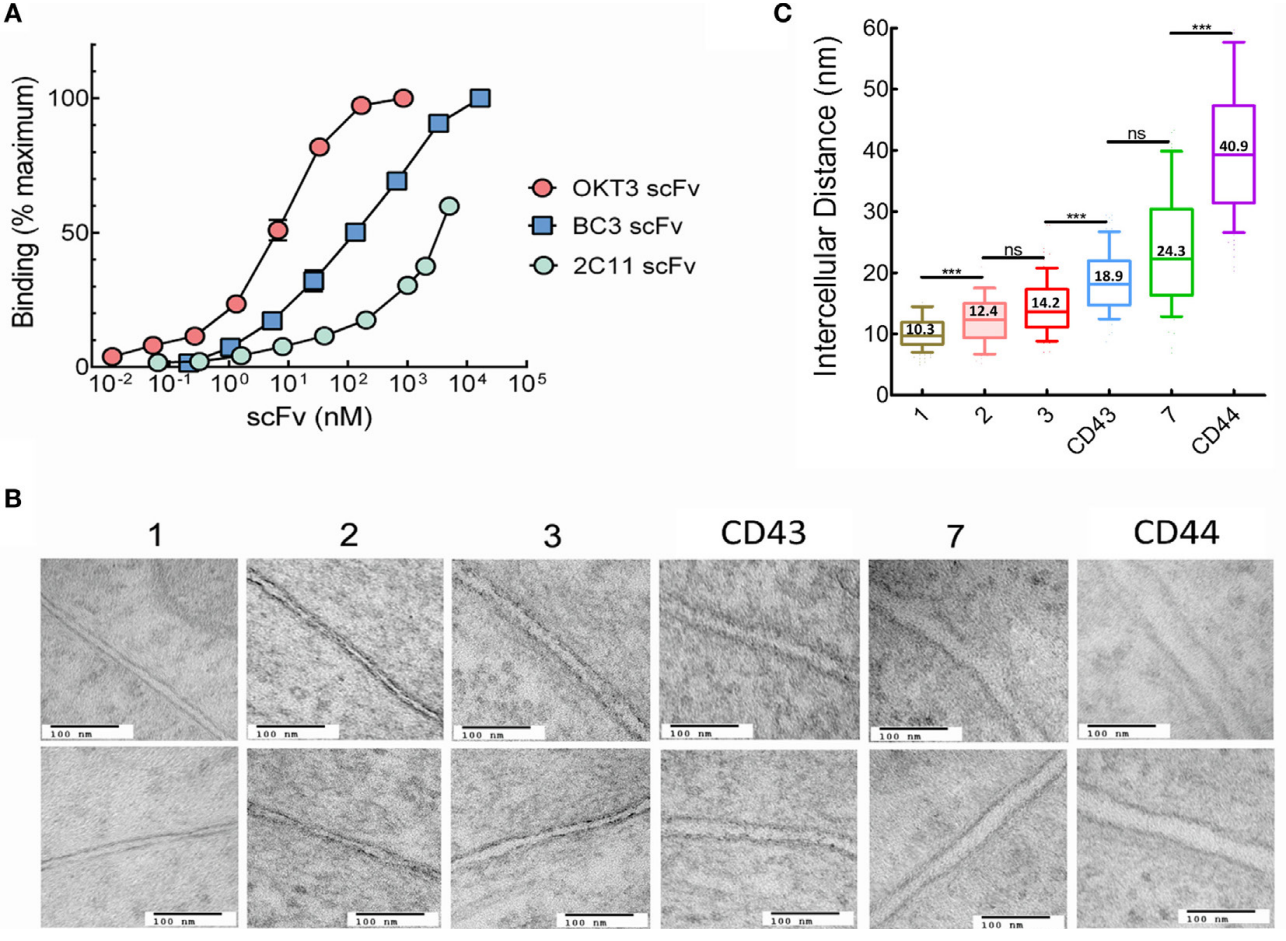
Intercellular distances between T cells and 3T3 antigen-presenting cells (APCs) depends on tether dimensions. **(A)** Binding of serial dilutions of OKT3 scFv, BC3 scFv, or 2C11 scFv to Jurkat (OKT3 and BC3) or 2B4 (2C11) T cells was determined by ELISA. Results show scFv binding relative to maximum binding of the individual scFv. Maximum binding was estimated for 2C11 scFv because saturation was not reached even at the highest concentration (250 µg/ml) of 2C11-BGP scFv examined. **(B)** Images of the interphase between Jurkat and 3T3 cells expressing the indicated membrane-anchored OKT3 scFv (150,000× original magnification. Scale bars, 100 nm). **(C)** Estimated mean intercellular distance between Jurkat and 3T3 cells expressing OKT3 scFv. Intercellular distances were measured at 11–20 equally spaced distances from 9 to 15 independent T cell-APC conjugates for each tether (*n* = 15, 15, 10, 9, 10, and 10 for 3T3 cells expressing OKT3-1, OKT3-2, OKT3-3, OKT-CD43, OKT3-7, and OKT3-CD44, respectively). The box and whisker plot shows the mean intercellular distances, the 10–90 percentile (box) and the minimum and maximum values (whiskers). Significant differences of intercellular distances between 3T3 cells expressing OKT3 scFv with different tethers are indicated (***p < 0.0001).

Immobilized antibodies against CD3 are often used to activate T cells due to their ability to selectively bind the TCR complex. We previously reported that membrane-anchored anti-CD3 scFv with short tethers effectively activated T cells, whereas elongation of the tether resulted in progressive loss of T cell activation (14, 17), consistent with the KS model of T cell activation. The anti-CD3 scFv in our previous studies were constructed from the 145-2C11 (2C11) antibody, which is a hamster anti-mouse CD3ε antibody with a reported relatively low affinity (*K*_d_ ~ 7 × 10^−8^ M) (25, 26). We selected two additional anti-CD3 antibodies with higher affinities to investigate the relationship between TCR ligand affinity and T cell activation. BC3 is mouse anti-human CD3ε antibody with a reported dissociation constant *K*_d_ of 8 × 10^−9^ M (27, 28), whereas the affinity of OKT3, a mouse anti-human CD3ε antibody was reported to be higher with a *K*_d_ of 5 × 10^−10^ M (28, 29). The relative affinities of scFv generated from 2C11, BC3, and OKT3 antibodies confirmed that OKT3 scFv displayed the highest apparent affinity to CD3 on T cells followed by BC3 and then 2C11 (Figure 2A).

The anti-CD3 scFv were genetically linked to extracellular tethers and expressed on 3T3 fibroblasts as aAPCs. The tethers were derived from proteins with 1, 2, 3, or 7 immunoglobulin-like domains as well as the extracellular domains of the CD43 or CD44 receptors. 3T3 fibroblasts were selected as “clean” aAPCs due to the absence of many costimulatory and adhesion molecules that could complicate interpretation of the data (30–32). We also tethered a membrane scFv with specificity for the hapten 2-phenyl-4H-1,3-oxazol-5-one *via* a one Ig-like extracellular domain (phOx-1), as negative binding control. We used transmission electron microscopy to investigate how the different tethers affected the intercellular distance between Jurkat T cells bound to 3T3 cells expressing membrane-tethered OKT3 scFv. There was a clear trend of increased intercellular distances with increased tether size (Figure 2B). Quantification of multiple T cell-APC conjugates showed that the intercellular distance between the cells increased from 10.3 nm for OKT3-1 (OKT3 scFv tethered to cells *via* a 1 Ig-like tether) up to 40.9 nm for OKT3-CD44 (Figure 2C). We conclude that the dimensions of the tether used to anchor anti-CD3 scFv on cells can affect the intercellular distance between T cells and the 3T3 APCs at the ligation site.

### Membrane-Tethered OKT3 Anti-CD3 scFv Can Bind T Cells

**Figure 3:**
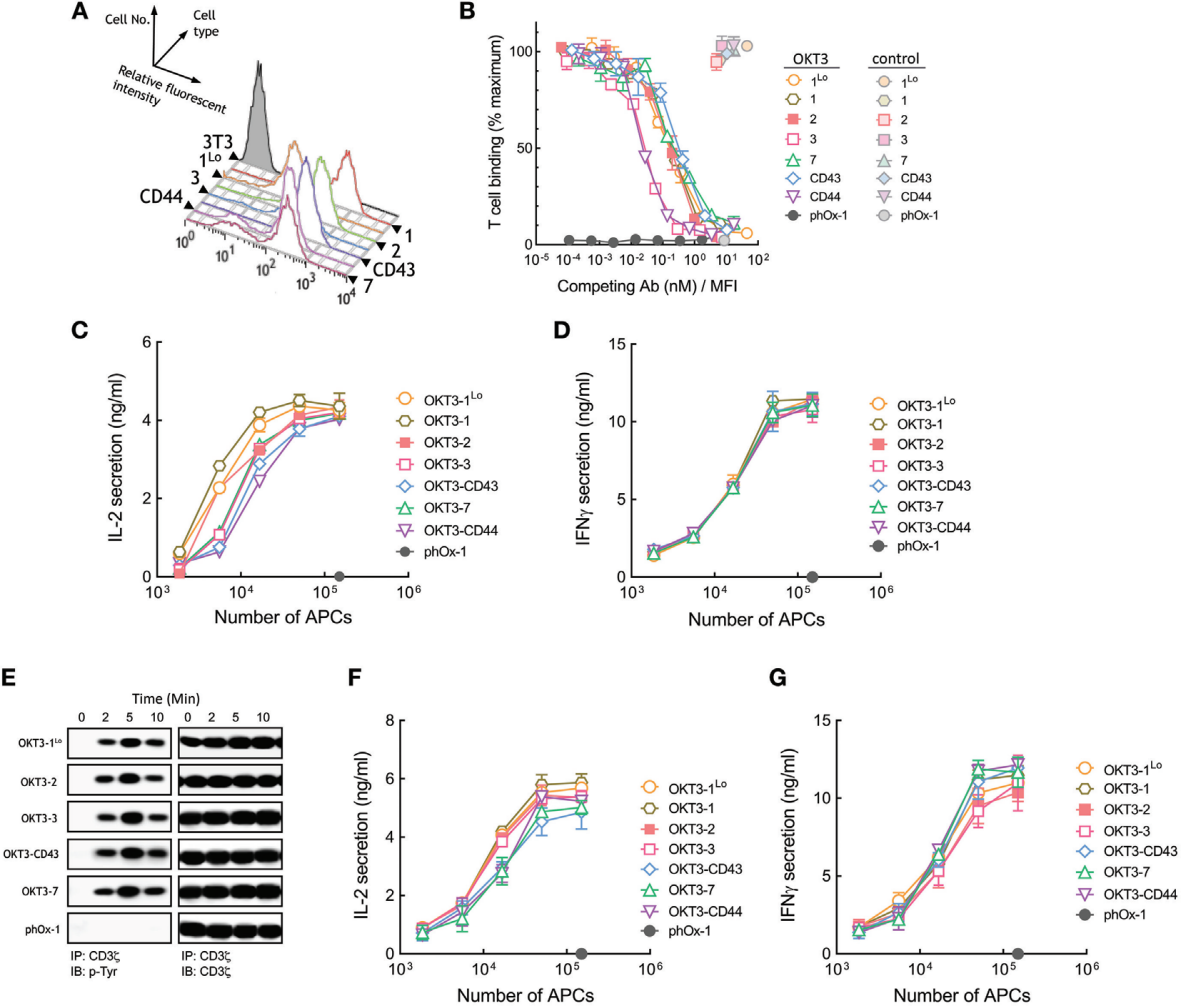
Membrane-tethered OKT3 scFv activates Jurkat T cells in a length-independent manner. **(A)** Surface expression of membrane-tethered OKT3 scFv on 3T3 cells was measured by FACS. OKT3-1^Lo^ cells express low levels of OKT3 scFv *via* the BGP tether (one Ig-like domain). **(B)** Calcein-AM-labeled Jurkat T cells were incubated with monolayers of 3T3 cells expressing membrane-tethered OKT3 scFv in the presence of serial dilutions of OKT3 IgG (dark symbols) or 10 µg/ml negative control IgG (light symbols). phOx-1 cells express a negative control scFv fused to the BGP tether. Unbound T cells were washed away and cellular fluorescence of the remaining bound cells was measured (*n* = 3). The *y*-axis shows the mean fluorescence of bound T cells, whereas the *x*-axis represents the concentration of soluble OKT3 IgG added normalized for the level of each membrane-tethered OKT scFv, as determined by mean immunofluorescence intensity of the 3T3 antigen-presenting cells (APCs) stained with anti-HA antibody. Bars, SD. **(C)** 5 × 10^4^ Jurkat T cells were incubated for 24 h with the indicated numbers of OKT3 APCs (OKT3-1, OKT3-1^Lo^, OKT3-2, OKT3-3, OKT3-7, OKT3-CD44, OKT3-C43) or control phOx-1 APCs before the concentration of IL2 **(C)** or IFN-γ **(D)** in the culture medium was determined (*n* = 3). Bars, SD. **(E)** 10^7^ Jurkat T cells were incubated with 10^6^ OKT3 APCs (OKT3-1^Lo^, OKT3-2, OKT3-3, OKT3-C43, OKT3-7) or control phOx-1 APCs for the indicated time before cells were lysed and CD3ζ was immunoprecipitated. Total CD3ζ (right panel) or phosphorylated CD3ζ (left panel) levels were determined by immunoblotting with anti-CD3ζ or anti-phosphotyrosine antibodies, respectively. 5 × 10^4^ naïve human T cells were incubated with the indicated number of OKT3 APCs for 24 h before the concentration of IL-2 **(F)** or IFN-γ **(G)** in the culture medium was determined (*n* = 3). Bars, SD.

3T3 APCs that stably express membrane-tethered OKT3 anti-CD3 antibodies were generated and sorted for similar surface expression by FACS using an antibody to detect the HA epitope tag present at the N-terminus of the scFv (Figure 3A). We also generated 3T3 cells that express about 10-fold lower levels of OKT3 scFv (OKT3-1^Lo^) to investigate the effects of ligand density on T cell activation. The functional binding activity of the membrane-tethered scFv was examined by measuring the retention of calcein-AM-labeled Jurkat T cells to the 3T3 APCs. APCs expressing membrane-tethered OKT3 anti-CD3 scFv bound T cells well, although antibodies anchored *via* the 1^Lo^ and 7 spacers bound fewer T cells than cells expressing OKT3 scFv anchored *via* the other tethers (Figure S1A in Supplementary Material). Minimal T cell binding was observed for unmodified 3T3 cells or 3T3 cells that expressed a membrane-anchored control antibody (phOx-1), demonstrating that binding depended on expression of anti-CD3 scFv. We also used a competition assay to confirm the influence of tether dimensions on T cell binding to minimize the effect of multivalent antibody binding to T cells. This assay measures binding of calcein-labeled T cells to 3T3 APCs in the presence of graded concentrations of soluble anti-CD3 antibodies. Soluble antibody concentrations were normalized to the level of membrane-tethered anti-CD3 scFv to take into account the different amounts of anti-CD3 scFv on the various cells. To validate the assay, we measured Jurkat T cell binding to APCs expressing high (OKT3-1) or low (OKT3-1^Lo^) levels of OKT3 scFv. As expected, more soluble OKT3 antibody was required to compete T cell binding to OKT3-1 APCs as compared to OKT3-1^Lo^ APCs (Figure S2A in Supplementary Material), even though both APCs expressed the identical anti-CD3 scFv on their surface. However, the competition curves were superimposable after soluble OKT3 concentrations were normalized for different expression levels of OKT3-1 on the APCs (Figure S2B in Supplementary Material), indicating that this competition binding assay can provide insight into relative affinities of membrane-tethered anti-CD3 scFv. Using the competition assay, we observed very similar binding of Jurkat T cells to APCs expressing OKT3-1^Lo^, OKT-1, OKT3-2, OKT3-7, and OKT3-CD43 but slightly lower binding of T cells to APCs expressing OKT3-CD44 and OKT3-3 (Figure 3B). Raw data of the MFI of T cell binding to APCs are displayed in Figure S3A in Supplementary Material. In general, there was no correlation between tether dimensions and the ability of the membrane-anchored OKT3 anti-CD3 scFv to bind T cells.

### Membrane OKT3 Anti-CD3 scFv Can Activate T Cells Regardless of Tether Dimensions

OKT3 APCs induced Jurkat cells to secrete IL-2 regardless of their dimensions (Figure 3C). Thus, OKT3 scFv that were anchored to 3T3 cells *via* longer tethers (OKT3-7, OKT3-CD43, and OKT-CD44) induced robust secretion of IL-2 from Jurkat T cells. APCs expressing either high or low levels of OKT3-1 (OKT3-1 and OKT3-1^Lo^, respectively) induced comparable T cell responses, demonstrating that ligand density is not the major factor influencing TCR activation by the antibodies. The OKT3 APCs also induced similar secretion of IFN-γ from Jurkat T cells (Figure 3D) regardless of the dimensions of the tether used to anchor OKT3 scFv on the cells. Measurement of tyrosine phosphorylation of CD3ζ in Jurkat T cells as a proximal measure of TCR triggering also demonstrated rapid CD3ζ phosphorylation regardless of the dimensions of membrane-anchored OKT3 antibodies (Figure 3E).

To rule out possible artifacts associated with the use of Jurkat T cells, we also examined the responses of purified human peripheral T cells to APCs expressing membrane-tethered OKT3 anti-CD3 scFv. In agreement with results obtained using Jurkat T cells, both short and elongated membrane-tethered OKT3 antibodies effectively induced secretion of IL-2 (Figure 3F) and IFN-γ (Figure 3G). Taken together, we conclude that membrane-anchored OKT3 scFv can trigger T cell activation regardless of the dimensions of the tether.

### T Cell Activation by Membrane-Anchored BC3 Anti-CD3 scFv Weakly Depends on Tether Dimensions

**Figure 4:**
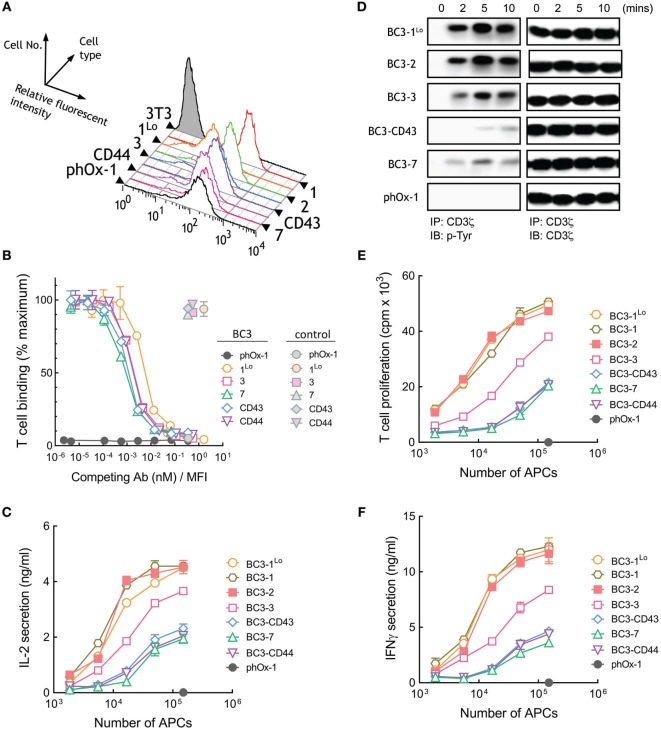
T cell activation by membrane-tethered BC3 scFv depends weakly on tether dimensions. **(A)** Surface expression of membrane-tethered BC3 scFv on 3T3 cells was measured by FACS. BC3-1^Lo^ cells express low levels of BC3 scFv *via* the BGP tether (one Ig-like domain). **(B)** Calcein-AM-labeled Jurkat T cells were incubated with monolayers of 3T3 cells expressing membrane-tethered BC3 scFv in the presence of serial dilutions of BC3 IgG (dark symbols) or 10 µg/ml negative control IgG (light symbols). Unbound T cells were washed away and cellular fluorescence of the remaining bound cells was measured (*n* = 3). The *y*-axis shows the mean fluorescence of bound T cells, whereas the *x*-axis represents the concentration of soluble BC3 IgG added normalized for the level of each membrane-tethered BC3 scFv, as determined by mean immunofluorescence intensity of the 3T3 antigen-presenting cells (APCs) stained with anti-HA antibody. Bars, SD. **(C)** 5 × 10^4^ Jurkat T cells were incubated with the indicated numbers of BC3 APCs or control phOx-1 APCs for 24 h before the concentration of IL-2 in the culture medium was determined (*n* = 3). Bars, SD. **(D)** 10^7^ Jurkat T cells were incubated with 10^6^ of BC3 APCs or control phox-1 APCs for the indicated times before cells were lysed and CD3ζ was immunoprecipitated. Total CD3ζ (right panel) or phosphorylated CD3ζ (left panel) levels were determined by immunoblotting with anti-CD3ζ or anti-phosphotyrosine antibodies, respectively. 5 × 10^4^ human T cells were incubated with BC3 APCs for 24 h before T cell proliferation **(E)** or IFN-γ in the culture medium **(F)** was determined (*n* = 3). Bars, SD.

A panel of 3T3 APCs that express similar levels of BC3 anti-CD3 scFv were generated (Figure 4A). As before, we also generated control APCs that express a membrane-tethered anti-phOx scFv that does not bind T cells as well as cells that express low levels of BC3-1 (BC3-1^Lo^). Similar number of Jurkat T cells bound to BC3 APCs (Figure S1B in Supplementary Material). Competition of T cell binding to BC3 APCs by soluble BC3 antibody revealed comparable affinities of BC3 scFv anchored to 3T3 cells *via* representative short and long tethers (Figure 4B). The raw mean fluorescence values for T cell binding to BC3 APCs also demonstrates similar binding of T cells by short and elongated BC3 scFv (Figure S3B in Supplementary Material). In contrast to APCs expressing membrane-tethered OKT3 scFv, we observed that elongation of the tether used to anchor BC3 scFv on the APCs resulted in attenuation of IL-2 secretion from Jurkat T cells (Figure 4C). Thus, APCs expressing BC3-7, BC3-CD43, and BC3-CD44 were about 10-fold less effective than BC3-1 at inducing IL-2 secretion from Jurkat cells. 3T3 APCs expressing either high (BC3-1) or low (BC3-1^Lo^) levels of BC3 scFv induced similar IL-2 secretion from T cells, demonstrating that the results are not sensitive to antibody density on the APCs. Examination of proximal TCR signaling also revealed that elongated BC3 scFv (BC3-CD43 and BC3-7) induced relatively weaker phosphorylation of CD3ζ in Jurkat T cells as compared to shorter BC3 scFv (BC3-1^Lo^ and BC3-2) (Figure 4D). Comparison of the ability of BC3 APCs to activate naive human T cells further confirmed that elongation of the tether used to anchor BC3 scFv on 3T3 cells lead to decreased proliferation (Figure 4E) and secretion of IFN-γ (Figure 4F) from the T cells. We conclude that in contrast to membrane-tethered OKT3 scFv antibodies, elongation of the tether used to anchor BC3 scFv on APCs resulted in attenuation but not abrogation of TCR activation.

### Membrane-Tethered 2C11 Activates T Cells in a Dimension-Dependent Manner

**Figure 5:**
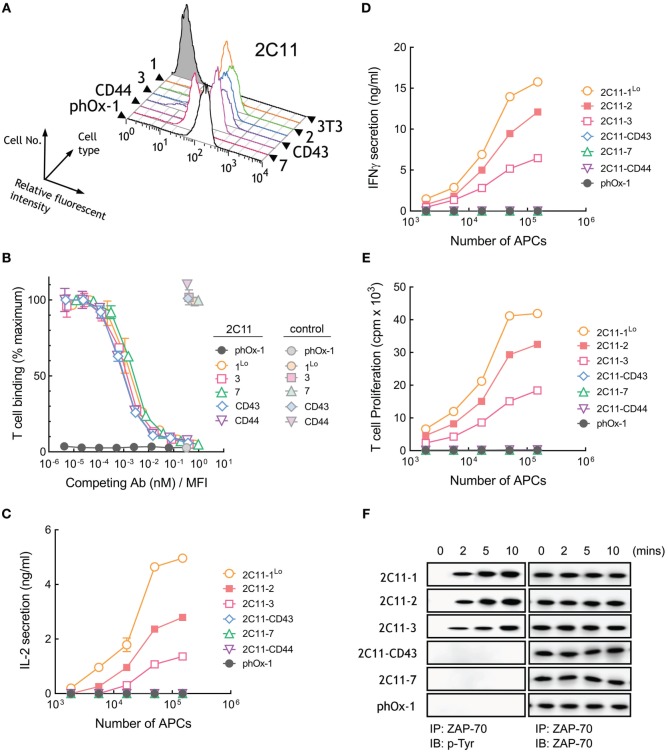
Membrane-tethered 2C11 scFv activate T cells in a dimension-dependent manner. **(A)** Surface expression of membrane-tethered 2C11 scFv on 3T3 cells was measured by FACS. **(B)** Calcein-AM-labeled naive mouse T cells were incubated with monolayers of 3T3 cells expressing membrane-tethered 2C11 scFv in the presence of serial dilutions of 2C11 IgG (dark symbols) or 10 µg/ml negative control IgG (light symbols). Unbound T cells were washed away and cellular fluorescence of the remaining bound cells was measured (*n* = 3). The *y*-axis shows the mean fluorescence of bound T cells, whereas the *x*-axis represents the concentration of soluble 2C11 IgG added normalized for the level of each membrane-tethered 2C11 scFv, as determined by mean immunofluorescence intensity of the 3T3 antigen-presenting cells (APCs) stained with anti-HA antibody. Bars, SD. **(C)** 5 × 10^4^ naïve mouse T cells were incubated with the indicated numbers of 2C11 APCs or control phOx-1 APCs for 24 h before measuring IL-2 **(C)** and IFN-γ **(D)** in the culture medium or the proliferation **(E)** of the T cells as assessed by the incorporation of ^3^H-thymidine (*n* = 3). Bars, SD. **(F)** 10^7^ B3Z cells were incubated with 10^6^ 2C11 APCs or control phOx-1 APCs for the indicated times before ZAP-70 was immunoprecipitated from cell lysates. Total ZAP-70 (right panel) or phosphorylated ZAP-70 (left panel) levels were determined by immunoblotting with anti-ZAP-70 or anti-phosphotyrosine antibodies, respectively.

A panel of 3T3 APCs that expressed similar levels of membrane-tethered 2C11 anti-CD3 scFv or lower levels of 2C11-1 (2C11-1^Lo^) were generated (Figure 5A) and demonstrated to bind to naive T cells with similar affinity (Figure S1C in Supplementary Material). Competition of T cell binding to 2C11 APCs by soluble 2C11 antibody confirmed comparable affinities of 2C11 scFv anchored to 3T3 cells regardless of the tether lengths (Figure 5B). Measurement of raw fluorescence signals of T cell binding to 2C11 APCs also demonstrated similar binding to cells expressing short and elongated 2C11 scFv (Figure S3C in Supplementary Material). 3T3 APCs expressing 2C11-1 effectively induced high levels of IL-2 secretion from T cells, whereas APCs expressing 2C11-2 and 2C11-3 were less effective (Figure 5C). Further elongation of membrane-tethered 2C11 scFv resulted in poor activation of T cells as IL-2 could not be detected in the culture medium of T cells incubated with 2C11-CD43, 2C11-7, or 2C11–CD44 APCs (Figure 5C). APCs that expressed elongated 2C11 scFv were also unable to induce the secretion of IFN-γ from T cells (Figure 5D) or induce T cell proliferation (Figure 5E). Tyrosine phosphorylation of ZAP-70, a proximal measure of TCR triggering, was also strongly dependent of tether length as shown for activation of B3Z mouse T cells with 3T3 APCs expressing membrane-anchored 2C11 scFv with no detectable phosphorylation of ZAP-70 observed for 3T3 APCs expressing 2C11-7 and 2C11-CD43 (Figure 5F). These results demonstrate that activation of T cells strongly depends on the dimensions of membrane-tethered 2C11 scFv, with complete loss of T cell activation by elongated 2C11 scFv.

### Elongated pMHC I and pMHC II Molecules Poorly Activate T Cells

**Figure 6:**
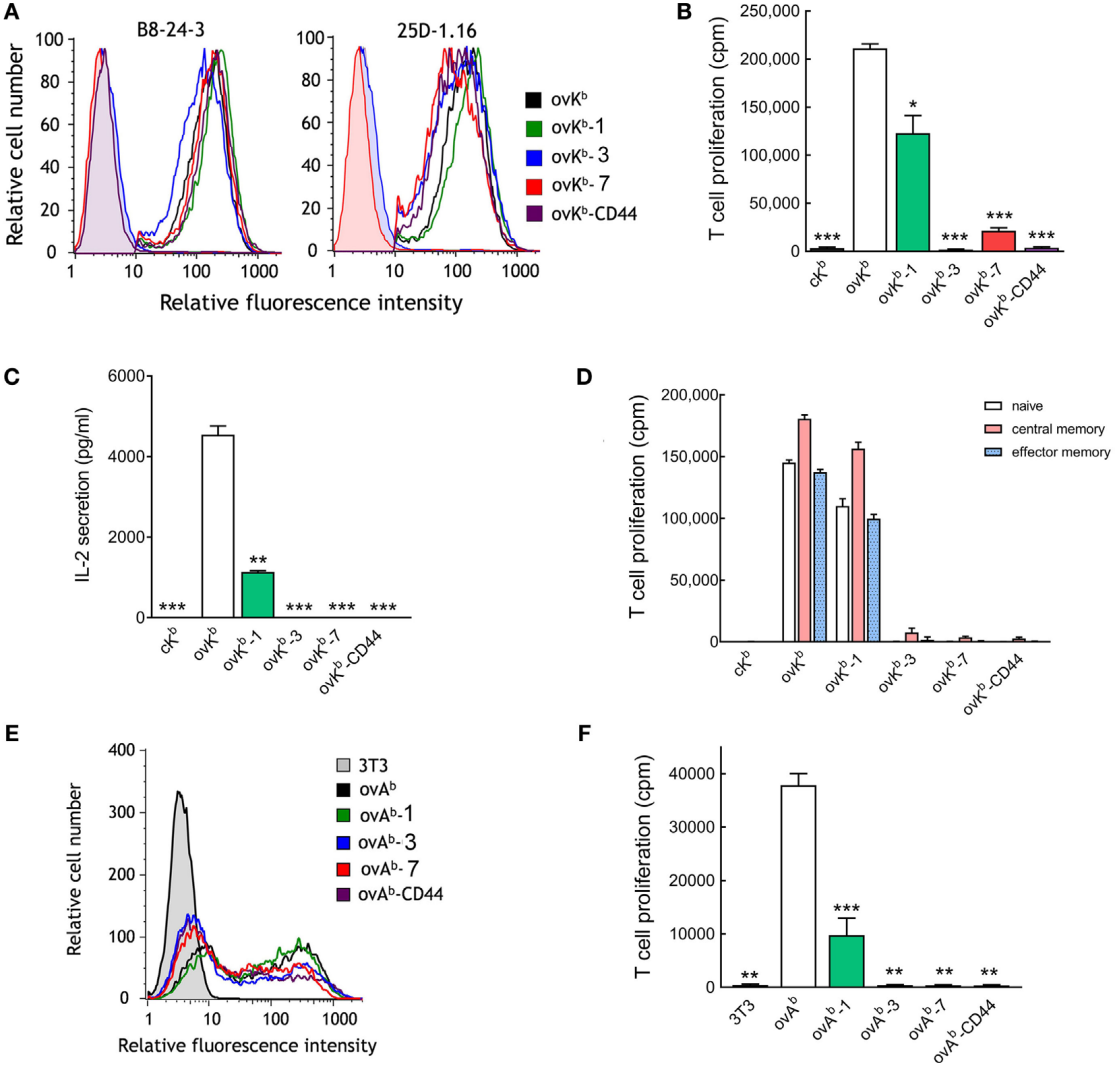
Effect of single-chain peptide/MHC on T cell activation. **(A)** 3T3 antigen-presenting cells (APCs) that stably express single-chain ovK^b^ molecules with different tethers were immunofluorescence stained for H-2K^b^ (PE-labeled AF6-88.5), SIINFEKL presented by H-2K^b^ (25D-1.16) or isotype controls (shaded curves) and analyzed on a flow cytometer. ^3^H-thymidine incorporation **(B)** and IL-2 secretion **(C)** in 2 × 10^5^ CD8^+^ T cells from OT-I mice incubated with 2 × 10^4^ ovK^b^ APCs for 48 h (*n* = 3). A single-chain MHC molecule (cK^b^) that presents the HPV 16 E7_48–55_ peptide was also expressed on 3T3 cells as a negative control. Bars, SD. Significant differences between ovK^b^ and other membrane-tethered ovK^b^ molecules are indicated: *p < 0.05, **p < 0.005, ***p < 0.0005. **(D)** The potency of ovK^b^ APCs to activate naïve, central memory, or effector memory OT-I T cells. **(E)** 3T3 APCs transiently expressing MHC class II single-chain ovA^b^ molecules were immunofluorescence stained with anti-FLAG M2 antibody and analyzed on a flow cytometer. **(F)** ^3^H-thymidine incorporation in 2 × 10^5^ OT-II CD4^+^ T cells stimulated with 2 × 10^4^ unmodified 3T3 cells or ovA^b^ APCs for 48 h (*n* = 3). Bars, SD. Significant differences between ovA^b^ and other membrane-tethered ovK^b^ molecules are indicated: **p < 0.005; ***p < 0.0005.

We observed a correlation of greater dependence of tether length on T cell activation as anti-CD3 scFv affinity decreased. We therefore examined the dependence on tether length for single-chain peptide-MHC molecules, which bind to TCRs with dissociation constants in the low micromolar range (33, 34). A single-chain peptide-MHC I molecule (ovK^b^) that presents a peptide derived from ovalbumin (OVA_257–264_) in the context of H-2K^b^ was generated and directly anchored on 3T3 fibroblasts (ovK^b^) or anchored *via* tethers with 1, 3, or 7 immunoglobulin-like domains (ovK^b^-1, ovK^b^-3, and ovK^b^-7) or *via* the extracellular domain of CD44 (ovK^b^-CD44). Similar levels of the ovK^b^ molecules were expressed on the surface of 3T3 fibroblasts as detected with an antibody against H-2K^b^ (Figure 6A, left panel). Similar expression of the scMHC molecules was also demonstrated by staining the cells with the 25D-1.16 antibody (Figure 6A, right panel), which recognizes H-2K^b^-OVA_257–264_ complexes (35). Importantly, we did not observe loss of binding accompanying increased scMHC length as measured by determining the fluorescence of calecien-stained OT-I T cells bound to monolayers of ovK^b^ APCs (Figure S4A in Supplementary Material). CD8^+^ T cells isolated from OT-I transgenic mice, which express a TCR that recognizes the OVA_257–264_ peptide in the context of H-2K^b^, were effectively activated by ovK^b^, while elongation by one Ig-like domain (ovK^b^-1) reduced the OT-I T cell proliferation to 60%. However, ovK^b^ anchored on 3T3 cells with tethers composed of 3 or 7 Ig-like domains (ovK^b^-3 and ovK^b^-7) or the CD44 extracellular domain (ovK^b^-CD44) were unable to induce robust proliferation of CD8^+^ OT-I T cells (Figure 6B). Likewise, ovK^b^ induced IL-2 secretion from OT-I T cells, but introduction of elongated tethers resulted in severe compromise of IL-2 secretion (Figure 6C). To investigate if specific T cell subsets respond differently to membrane-anchored ovK^b^ molecules, OT-I T cells were separated into naive, central memory, or effector memory population by FACS and then stimulated with identical numbers of 3T3 ovK^b^ APCs. Activation of all populations of OT-I T cells displayed strong dependence on the dimensions of the ovK^b^ tether with strong proliferation induced with ovK^b^ and ovK^b^-1 but poor proliferation induced by ovK^b^-3, ovK^b^-7, and ovK^b^-CD44 (Figure 6D).

A single-chain peptide-MHC II molecule (ovA^b^) that presents a peptide derived from ovalbumin (OVA_323–339_) in the context of I-A^b^ with tethers containing 0, 1, 3, or 7 immunoglobulin-like domains (ovA^b^, ovA^b^-1, ovA^b^-3, and ovA^b^-7) or the extracellular domain of CD44 (ovA^b^-CD44) were generated. Similar levels of the ovA^b^ molecules were expressed on the surface of transiently transfected 3T3 fibroblasts as detected with an antibody against a FLAG M2 epitope present in the ovA^b^ molecule (Figure 6E). There was no obvious difference in the number of OT-II T cells bound to monolayers of ovA^b^ APCs as measured by fluorescence of calcerin AM-stained T cells (Figure S4B in Supplementary Material). 3T3 APCs expressing ovA^b^ most effectively activated OT-II CD4^+^ T cells while ovA^b^-1 was significantly less effective and ovA^b^ anchored to the cells *via* more elongated tethers poorly activated OT-II T cells (Figure 6F). We conclude that activation of T cells by pMHC strongly depends on the physical dimensions of the complex.

### Elongated Membrane-Anchored Low-Affinity OKT3 scFv Poorly Activate T Cells

**Figure 7:**
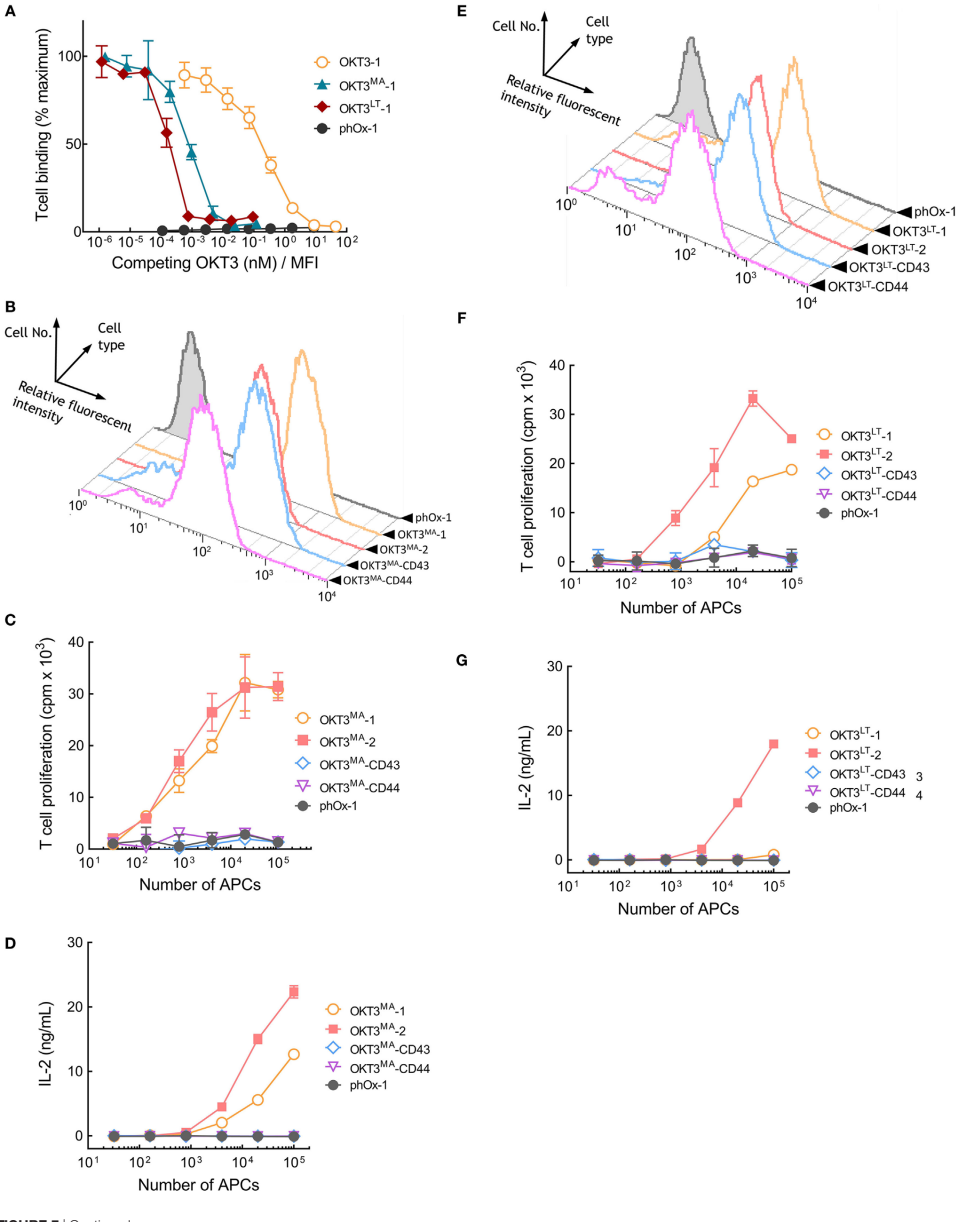
Membrane-tethered low-affinity OKT3 scFv activate T cells in a dimension-dependent manner. **(A)** Calcein-AM-labeled Jurkat T cells were incubated with monolayers of 3T3 antigen-presenting cells (APCs) expressing membrane-tethered OKT3 scFv (OKT3-1) or OKT3 variant scFv (OKT3^MA^-1 and OKT3^LT^-1) in the presence of defined concentration of soluble OKT3 IgG. The *y*-axis shows the mean fluorescence (*n* = 3) of bound T cells, whereas the *x*-axis represents the concentration of soluble OKT3 antibody added normalized for the level of each membrane-tethered scFv, as determined by mean immunofluorescence intensity of the 3T3 APCs stained with anti-HA antibody. Bars, SD. **(B)** The expression of membrane-tethered OKT3^MA^ scFv on 3T3 APCs was determined by FACS. The proliferation as measured by the incorporation of ^3^H-thymidine into cellular DNA of the T cells **(C)** and IL-2 secretion **(D)** of 10^5^ human peripheral T cells incubated for 48 h with the indicated numbers of OKT3^MA^ or control phOx-1 APCs (*n* = 3), Bars, SD. **(E)** FACS of 3T3 APCs expressing membrane-tethered OKT3^LT^ or control phOx-1 scFv. The proliferation as measured by the incorporation of ^3^H-thymidine into cellular DNA of the T cells **(F)** and IL-2 secretion **(G)** of 10^5^ human peripheral T cells incubated for 48 h with the indicated numbers of OKT3^LT^ or control phOx-1 APCs (*n* = 3), Bars, SD.

Our results reveal a correlation between the affinity of TCR ligands and the effect of tether dimensions on their ability to activate T cells. Thus, elongated high-affinity OKT3 scFv effectively activate T cells, whereas low-affinity pMHC rapidly loss their ability to activate T cells as tether length increases. We sought to directly test the influence of TCR ligand affinity on T cell activation by creating OKT3 scFv molecules with reduced binding affinity. A library of membrane-anchored OKT3 scFv variants with all possible amino acids at two amino acid positions (R55 and Y57) that contact CD3ε (23, 24) was screened. Two OKT3 variants (OKT3^MA^ and OKT3^LT^) possessing R55M, Y57A and R55L, Y57T amino acid substitutions and displaying reduced binding to Jurkat T cells were selected. The relative affinities of membrane-anchored OKT3-1, OKT3^MA^-1, and OKT3^LT^-1 scFv were determined by adding soluble OKT3 IgG to compete T cell binding to the APCs. The normalized concentration of soluble OKT3 IgG required to reduce Jurkat T cell binding by 50% to APCs expressing OKT3-1 was 0.23 ± 4.9 × 10^−3^ nM/MFI (Figure 7A). By contrast, only 9.1 × 10^−4^ ± 1.1 × 10^−4^ and 2.2 × 10^−4^ ± 8.8 × 10^−5^ nM/MFI soluble OKT3 IgG was required to compete 50% T cell binding to APCs expressing OKT3^MA^-1 or OKT3^LT^-1, respectively (Figure 7A), corresponding to about 250-fold and 1,000-fold lower apparent binding affinities as compared to the parental OKT3-1.

The OKT3^MA^ scFv was stably expressed on 3T3 APCs *via* different tethers. Flow cytometer analysis showed similar levels of OKT3^MA^-1, OKT3^MA^-2, and OKT3^MA^-CD43 but somewhat lower levels of OKT3^MA^-CD44 were present on the APCs (Figure 7B). OKT3^MA^-1 and OKT3^MA^-2 effectively activated naïve human peripheral T cells as determined by T cell proliferation (Figure 7C) and IL-2 secretion (Figure 7D). In contrast to APCs expressing wild-type OKT3 scFv (Figure S5 in Supplementary Material), OKT3^MA^-CD43 and OKT3^MA^-CD44 were unable to activate T cells (Figures 7C,D). T cell activation was insensitive to the levels of membrane-anchored OKT3^MA^-1 (Figure S6 in Supplementary Material), indicating that variations in T cell activation are unlikely caused by differences in the levels of scFv on 3T3 APCs.

The lower affinity OKT3^LT^ variant of the OKT3 scFv was also expressed on 3T3 APCs *via* different tethers (Figure 7E). APCs that expressed OKT3^LT^-1 or OKT3^LT^-2 induced proliferation of naïve human peripheral T cells, whereas APCs expressing OKT3^LT^-CD43 or OKT^3LT^-CD44 did not effectively activate T cells (Figure 7F). Of note, APCs expressing OKT3^LT^-2 activated T cells better than OKT3^LT^-1. APCs expressing OKT3^LT^-2 also induced IL-2 secretion from T cells, whereas OKT3^LT^ anchored on APCs with more elongated tethers did not induce IL-2 secretion (Figure 7G). Surprisingly, OKT^LT^-1 on APCs did not effectively induce IL-2 secretion from T cells. We conclude that reduction of the affinity of OKT3 results in a dramatic shift from dimension-independent to dimension-dependent activation of T cells by membrane-anchored anti-CD3 antibodies.

### Surface Density Is Not the Major Factor Driving the Dimension-Independent T Cell Activation by Membrane-Tethered OKT3 Anti-CD3 scFv

**Figure 8:**
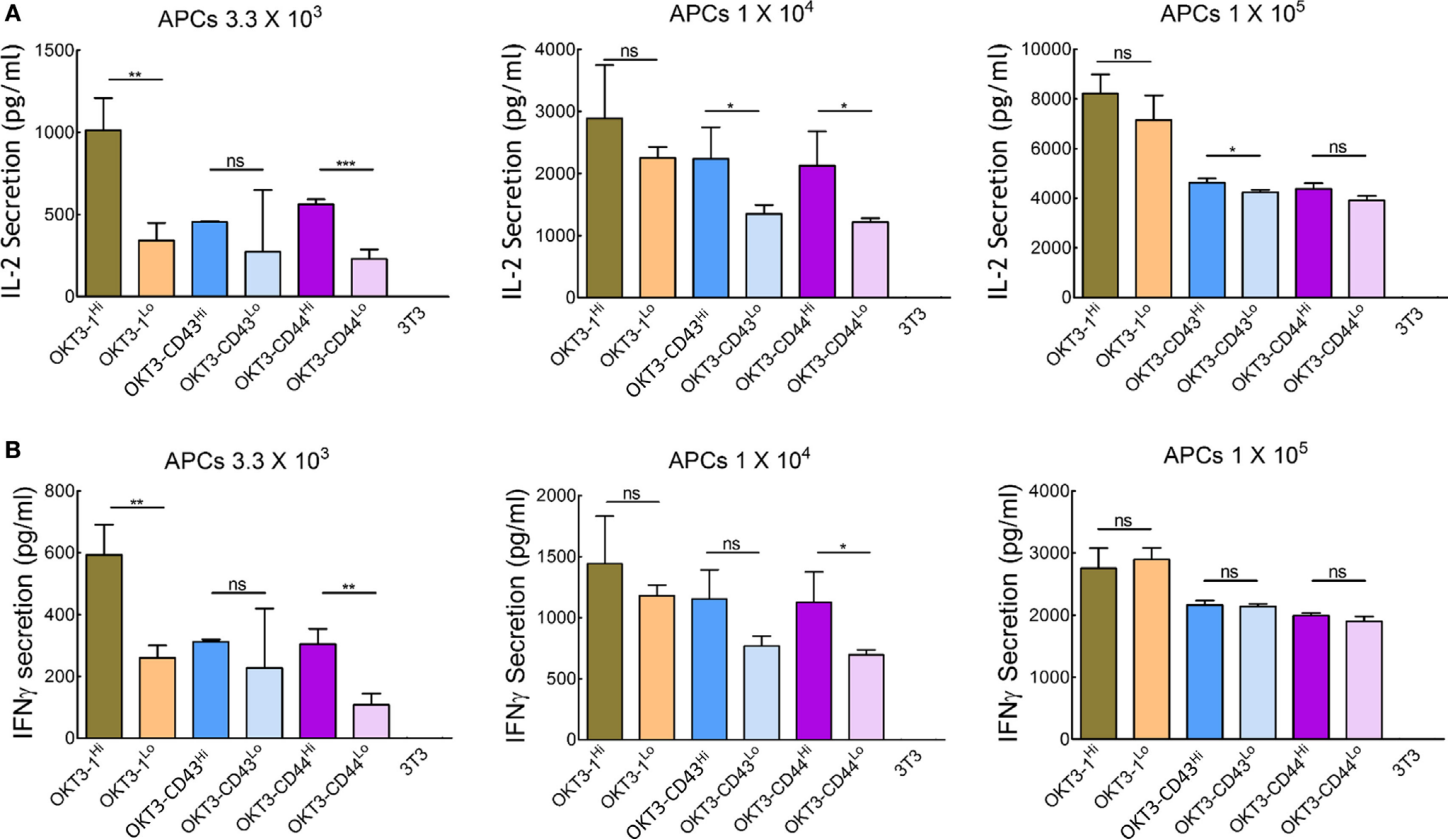
Activation of Jurkat T cells by 3T3 antigen-presenting cells (APCs) expressing different densities of membrane-tethered OKT3 scFv. IL-2 **(A)** and IFN-γ **(B)** secretion from 1 × 10^5^ Jurkat T cells incubated with 3.3 × 10^3^ (left panel), 1 × 10^4^ (middle panel), or 1 × 10^5^ (right panel) OKT3 APCs expressing high or low densities of OKT3-1, OKT3-CD43, or OKT3-CD44 are shown. The number of OKT3 ligands on 3T3 cells is shown in Figure S7 in Supplementary Material. Cytokine secretion was measured by ELISA 48 h after T cell activation (*n* = 3). Bars, SD.

We further investigated the effect of the surface density of high-affinity ligands on APCs to determine if saturation of TCR triggering occurred. Stable 3T3 cells were sorted for low (~10 ligands per μm^2^) or high (~110–180 ligands per μm^2^, similar to most of the APCs used in our study) expression of OKT3-1, OKT3-CD43, or OKT3-CD44 (Figure S7 in Supplementary Material). Comparison of the ability of these cells to trigger cytokine secretion from Jurkat T cells showed that cells expressing high and low ligand densities induced similar IL-2 secretion (Figure 8A) and IFN-γ secretion (Figure 8B) at high ratios of APCs:T cells. At lower ratios of APCs:T cells, T cells could be activated, although to a reduced level, by both short and elongated ligands, indicating elongated high-affinity ligands can effectively activate T cells even at low surface densities (Figure 8). However, it is possible that different results would be observed at even lower surface densities of the ligands.

## Discussion

The mechanism by which engagement of TCRs first triggers signaling remains unclear. Here, we used a series of membrane-tethered anti-CD3 antibodies and pMHC molecules to investigate systemically how receptor affinity and topology alter TCR triggering. We show that TCR signaling strongly depends on the dimensions of low-affinity but not high-affinity membrane-anchored ligands. Thus, elongation of the extracellular dimensions of pMHC molecules, which display relatively low affinities for TCRs, results in loss of TCR triggering. Likewise, elongation of the dimensions of a low-affinity membrane-tethered anti-CD3 scFv antibody (2C11) caused progressive inability to induce TCR phosphorylation, cytokine secretion, and T cell proliferation. By contrast, elongation of the dimensions of a higher affinity anti-CD3 scFv (BC3) did not totally block TCR triggering and a high affinity membrane-tethered anti-CD3 scFv (OKT3) triggered TCR signaling, proliferation, and cytokine secretion regardless of its dimensions. Reducing the affinity of OKT3 scFv by 250-fold (OKT3^MA^) or 1,000-fold (OKT3^LT^) converted this ligand from a dimension-independent to dimension-dependent forms, with TCR triggering observed only for the short but not elongated membrane-anchored anti-CD3 scFv. Taken together, our results reveal that in contrast to previous studies (11, 13, 36), elongation of membrane-anchored TCR ligands does not universally attenuate T cell activation. Rather, the effectiveness of TCR triggering correlates with ligand dimensions for “low” affinity ligands, whereas high-affinity ligands can effectively activate T cells regardless of their membrane topology.

Several investigations have reported an inverse correlation between the physical dimensions and the effectiveness of TCR ligands in inducing T cell activation (11, 13, 36, 37). These observations are consistent with the KS model that suggests that the relatively small intercellular distance between APCs and T cells at the ligation site is required to physically segregate large receptor protein tyrosine phosphatases away from engaged TCR/pMHC complexes, resulting in a shift to CD3 ITAM phosphorylation. Elongation of TCR ligands is therefore predicted to remove steric barriers, allowing more close proximity between receptor protein tyrosine phosphatases and engaged TCR complexes, thereby maintaining TCR ITAMs in an inactive unphosphorylated state. Previous studies have demonstrated that elongation of membrane-tethered pMHC molecules (SCTs) on artificial APCs progressively reduced IL-2 secretion from mouse and human T cells (11, 13). We also previously observed that elongation of membrane-tethered 2C11 anti-CD3 scFv on fibroblasts prevented T cell proliferation and cytokine secretion (12, 14). Other evidence demonstrating an important role for CD45 segregation in T cell activation includes studies demonstrating segregation of CD45 from the site of triggered TCRs (38–40), reconstituted systems showing that CD45 is critical for maintaining the TCR/CD3 complex in a dephosphorylated state (41, 42) and studies showing that truncation of the CD45 ectodomain abrogates segregation and/or TCR triggering (37, 42, 43).

In the present study, we also observed that APCs expressing elongated membrane-tethered 2C11 scFv and pMHC molecules (i.e., elongated with CD43, CD44, and 7 Ig-like domains) were unable to effectively induce CD3 ITAM phosphorylation, T cell proliferation, and cytokine secretion. However, APCs expressing membrane-tethered BC3 and OKT3 scFv elongated with the identical tethers (CD43, CD44, and 7 Ig-like domains) and similar surface densities of ligands induced phosphorylation of CD3ζ and activated both Jurkat T cells and naïve human T cells. Since the dimensions of elongated membrane-anchored BC3 and OKT3 scFv are identical to the elongated 2C11 scFv and pMHC molecules, topological segregation of receptor protein tyrosine phosphatases from engaged TCRs may be a result of size-based segregation of surface receptors with different sizes, as observed during formation of the central supramolecular activation cluster (cSMAC), rather than the major trigger that initiates TCR signaling. However, we did not directly examine CD45 segregation in our study. Recently, Vale and his colleagues showed that the negatively charged CD45 cytoplasmic domain is excluded from phosphorylated LAT clusters that favor inclusion of positively charged proteins, indicating that CD45 segregation is independent of the external CD45 domain (44). CD45 was also found to be constitutively excluded from TCR microclusters present on unstimulated T cells in the absence of antigen stimulation (45). It is possible that segregation of CD45 from engaged TCRs is required for prolonged and robust signaling but more studies are required to address this issue. Our results suggest that other explanations besides CD45 segregation may be needed to fully describe the dependence of T cell activation on ligand dimensions.

We find that the dependence of TCR triggering on membrane anti-CD3 antibody topology correlates with the affinity of ligand-TCR interactions. The affinities of the anti-CD3 scFv can be roughly ranked as OKT3 > BC3 > 2C11 > OKT3^MA^ >OKT3^LT^. Competition experiments demonstrated that OKT3^LT^ displays about 1,000-fold lower affinity as compared to OKT3. Membrane-tethered ovK^b^ presenting OVA_257–264_ polypeptide and membrane-tethered ovA^b^ presenting OVA_323–339_ polypeptide display low micromolar affinities to CD8^+^ OT-I and CD4^+^ OT-II mouse T cells, respectively (33, 46, 47), which are predicted to be near the affinity of OKT3^LT^ scFv. Comparison of T cell activation by ligands anchored *via* an intermediately sized three immunoglobulin-like tether showed that OKT3-3 potently activates T cells, BC3-3 displays slightly reduced activation, 2C11-3 clearly activates T cells but at a much reduced level while ovK^b^-3 and ovA^b^-3 were incapable of activating T cells. Likewise, comparison of ligands anchored *via* very long tethers (i.e., CD44) demonstrated that high affinity OKT3-CD44 activated T cells almost as well as OKT3-1, moderate affinity BC3-CD44 could induce about half maximal T cell activation whereas low-affinity 2C11-CD44 could not activate T cells. Importantly, a large reduction of the affinity of OKT3 (OKT3^MA^ and OKT3^LT^) restored the dependence of T cell activation on the length of the membrane tether (i.e., OKT3^MA^-CD44 and OKT3^LT^-CD44 could not activate T cells), demonstrating that our observations were not due to differences in the behavior of mouse and human T cells. Taken together, our results indicate that the dependence of TCR triggering on ligand dimensions is strong for low-affinity ligands but is much weaker for high-affinity ligands.

We tethered TCR ligands on 3T3 cells by fusion to the murine B7.1 transmembrane domain and cytoplasmic tail. This may raise a question of whether our ligands are expressed as dimers as found for native B7.1. However, previous studies have shown that dimerization of B7.1 occurs due to interactions between amino acids present in the extracellular domains of B7.1. Ikemizu and colleagues showed that a soluble form of human B7.1 (amino acids 1–201), which encompasses most of the extracellular domain, forms dimers in solution, and that the hydrophobic residues V11, V22, G45, M47, I58, D60, T62, and L70 are mostly responsible for such dimerization (48). Moreover, mutating amino acids L58 or I68 in murine B7.1 results in formation of B7.1 monomers (49). Our membrane-tethered constructs encode a B7.1 polypeptide starting at P237 and ending at the stop codon and therefore do not encompass the extracellular domain of B7.1. We showed previously that proteins could be expressed on the surface of cells in monomeric form when using the B7.1 transmembrane and cytoplasmic domains (19). The B7.1 cytoplasmic tail is also critical for protein transportation and correct display of chimeric proteins and ligands on the cell surface (50).

We investigated the possibility that TCR triggering is saturated by high surface densities of high-affinity OKT3 scFv (110–180 per µm^2^) by titrating expression to a lower surface density (~10 per µm^2^). However, both high and low surface densities of elongated OKT3 scFv triggered T cell activation. We also titrated the numbers of APCs in most experiments, which should reduce saturation of T cell responses. Our data using low-affinity ligands (Figures 5–7) are consistent with previous reports in which ligand elongation dramatically decreased TCR triggering (11–14). The clearly observed trend of decreased T cell triggering by elongated low-affinity ligands demonstrates that we were not operating at a ligand density that obscured this expected trend. However, we cannot rule out the possibility that elongated high-affinity ligands would be unable to activate T cells at lower surface densities (<10 ligands per μm^2^).

One explanation for decreased T cell activation by elongated ligands is that the longer tethers may be flexible so that anti-CD3 scFv can occupy a much larger conformational space than ligands anchored with shorter tethers. This would be expected to decrease the probability of ligand contact and bond formation with TCRs, thereby lowering their effective binding affinities. Indeed, it has been shown that extending low-affinity adhesion molecules (CD48) with longer tethers causes a 10-fold increase in the density of the ligands needed to mediate adhesion to CD2 (51). However, we did not observe any trend between apparent binding affinity and ligand dimensions in assays that measured raw fluorescence of bound cells, in studies using serially diluted soluble anti-CD3 antibodies to compete binding of T cells to APCs expressing membrane-tethered anti-CD3 scFv, or after normalization of binding data to take into account differences in membrane surface densities of the ligands. These results suggest that anti-CD3 scFv ligands displayed comparable binding affinities over the tether lengths investigated in our study. We do not know why increased tether length affects CD48 binding to CD2, but it is possible that the orientation of the intermolecular binding interface is altered or perhaps is related to the exceptionally low-affinity interaction between CD2 and CD48 (*K*_d_ ~ 10^−4^ M) (52), which is much lower that the affinities of the ligands examined in our study.

Our results are consistent with an increasing number of independent studies, including those from our lab, demonstrating that mechanical forces acting on TCRs can specifically trigger T cell activation (14, 53–58). Mechanical forces acting on TCRs during ligand engagement may be transmitted inside T cells *via* conformational changes in a conserved core structure of the transmembrane domains of the TCR alpha/beta chains, which organizes the TCR complex into an intimately packed eight-helix bundle (59). The cytoplasmic tails of CD3ε and CD3ζ subunits of the TCR complex bind to the inner leaflet of the lipid bilayer but undergo conformational changes that dissociate the cytoplasmic domains from the membrane during TCR engagement (60, 61). *In situ* proximity assays have shown that the cytosolic tails of the CD3ζζ subunits physically move together during TCR engagement (62). CD3ζζ subunits may pivot around a hinge formed between positively charged amino acids in the TCR alpha chain transmembrane domain and negatively charged amino acids in the transmembrane domains of the CD3ζζ subunits; mechanical forces acting on the TCR may thus act to swing the cytosolic domains of the CD3ζζ subunits together for initiation of signal transduction (62). The intracellular conformational changes in engaged TCR complexes allow Src kinase signal transduction that ultimately leads to T cell proliferation and activation of effector functions.

Although several labs have now found that TCRs can be triggered to signal in response to mechanical forces, the source of mechanical force to trigger TCRs is still undefined (55, 63, 64). Based on our results, we propose that there are at least two possible sources of forces acting on engaged TCRs: (1) movement of engaged TCRs relative to CD3 in the plasma membrane to facilitate productive engagement between short TCR/pMHC molecules in an environment of neighboring large extracellular membrane proteins and adhesion molecules such as CD45, ICAM-1, LFA-1, and CD43 that hinder close contact between opposing T cell and APC plasma membranes (65) [reviewed in Ref. (7, 66)] and (2) thermally induced stochastic membrane fluctuations (67–69).

Movement of TCRs relative to CD3 may occur when pMHC and TCR alpha/beta chains are engaged because they span a relatively small distance (~15 nm) as compared to other larger surface receptors (70). The fixed intercellular distance between APC/T cell membranes at sites of productively engaged pMHC and TCRs may cause a tensive force on engaged TCRs to accommodate surrounding larger receptor proteins (Figure S8A in Supplementary Material). Indeed, normal function and signaling *via* TCR microclusters requires that the microclusters are closely surrounded by a ring of large adhesion molecules, including LFA-1 and ICAM-1 (71, 72). LFA-1/ICAM-1 interactions have been shown to be critically important for T cell activation independent of their adhesive function (73). We expect that differences in the sizes of engaged TCRs and nearby adhesion molecules and surface receptors might be the normal physiological source of force acting on productively engaged pMHC-TCR. Thus, low-affinity ligands such as pMHC become ineffective stimulators as their dimensions increase and there is less disparity in their dimensions as opposed to surrounding adhesion proteins and receptors (Figure S8B in Supplementary Material). Such a force would be almost instantaneous, which could account for rapid TCR triggering, which occurs within seconds of TCR engagement (74). Size-related tensive forces may also be consistent with continual and sustained TCR activation in the distal and peripheral supramolecular activation cluster where mechanical forces might be generated on engaged TCRs to accommodate the abundant surrounding large receptors and adhesion molecules in these areas of T cells (40, 75). This model may also help explain why signaling by engaged TCRs in the cSMAC is relatively downregulated even though there are abundant and large clusters of engaged TCRs in the cSMAC; the sparsity of large surface receptors and adhesion molecules in the mature cSMAC cannot provide tensive forces on engaged TCRs since receptors and ligands in the mature cSMAC are of similar dimensions (76).

**Figure 9:**
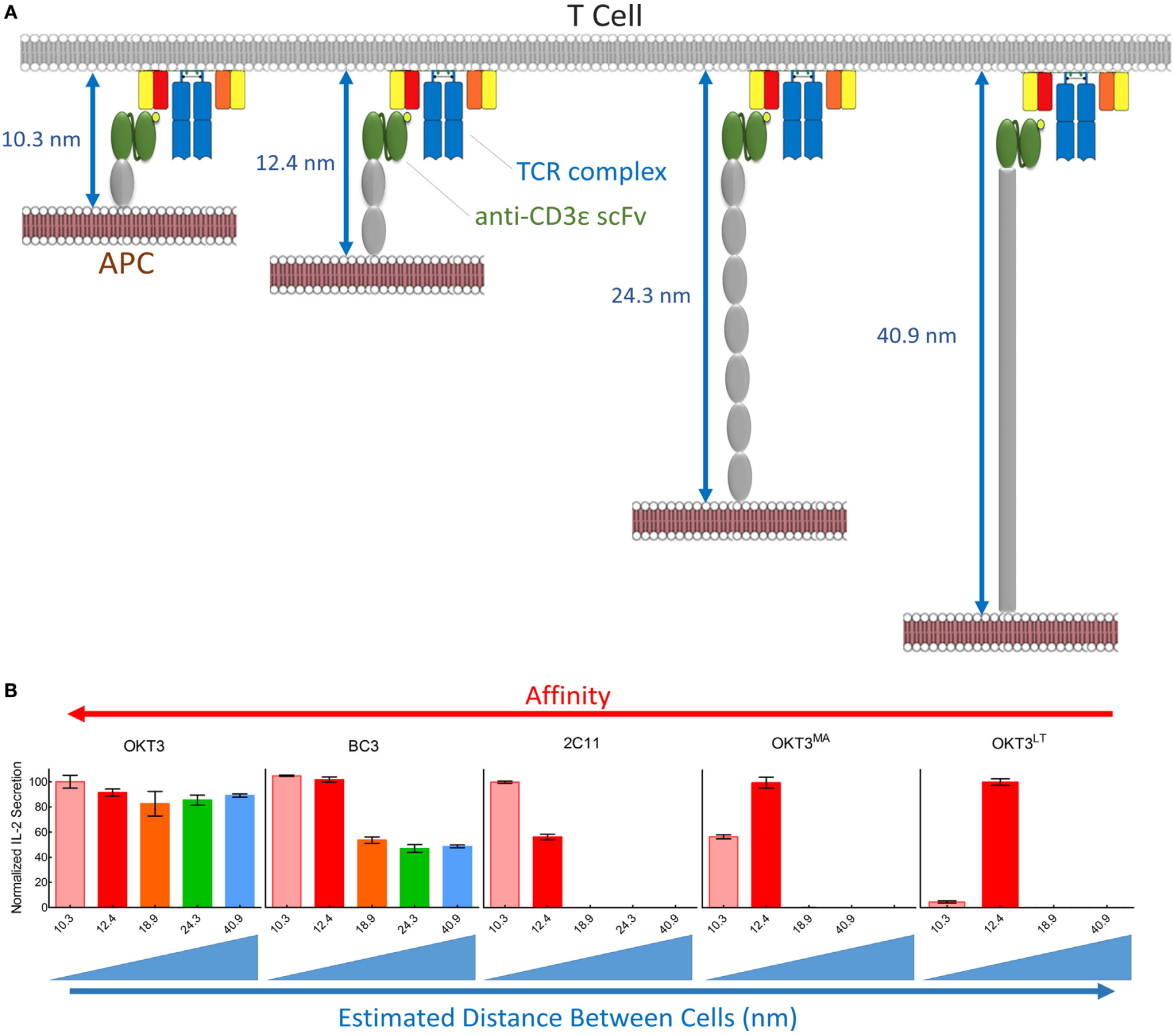
Summary of T cell receptor (TCR) triggering by membrane-tethered anti-CD3 scFv. **(A)** Schematic diagram showing the estimated distance between antigen-presenting cell (APC) and T cells upon scFv/TCR engagement. **(B)** Normalized values of IL-2 secretion among five membrane-tethered scFv we used in this study arranged from the highest (left) to the lowest (right) affinity. The relationship between triggering and interspatial distance between APC and T cells is also indicated.

We speculate that an additional force may be generated by non-physiologically high-affinity ligands where TCRs are engaged for an abnormally prolonged time. Many studies have shown that the plasma membrane of cells, including lymphocytes, display thermally induced stochastic displacements or fluctuations with an amplitude of several tens of nanometers at frequencies of 0.1–1 s (67, 68, 77, 78). We therefore speculate that mechanical forces can be generated, albeit inefficiently, by stochastic membrane fluctuations (sometimes the opposing T cell and APC membranes at sites of TCR engagement are fluctuating in the same direction and sometimes they move in opposite directions; forces should only be generated when they move in opposite directions). This fits with recent findings that frequently applied serially forces acting on the TCR can be summed for effective activation of CD8^+^ T cells (79). We expect that this phenonomen is non-physiological because only high-affinity ligands remain engaged for sufficient time to generate a triggering force by this inefficient method, while low-affinity ligands will rupture binding before triggering occurs (55). We envision that for very high-affinity ligands, such as OKT3, summation of mechanical forces generated by stochastic membrane fluctuations is sufficient for full TCR triggering, whereas for an intermediate affinity ligand such as BC3, TCR engagement may be sufficiently long to only generate partial T cell activation by stochastic membrane fluctuations. Thus, BC3 scFv shows partial length dependence. As tether length decreases, there is an additional contribution from physiological activation (the first mechanism of mechanical force generation). For low-affinity ligands (i.e., pMHC and 2C11 scFv), there may be insufficient binding time to accumulate triggering forces by membrane fluctuations, and force is only generated by the first mechanism, which is strictly dependent on ligand dimensions (Figure S9 in Supplementary Material). Indeed, we observed that elongated low-affinity ligands (pMHC, 2C11, and OKT3 mutants) did not activate T cells, while BC3 scFv produced about 30% maximal T cell activation and the higher affinity OKT3 scFv could maximally activate T cells regardless of tether dimensions (Figure 9; Table S1 in Supplementary Material). These results suggest that TCR activation *via* forces generated by stochastic membrane fluctuations may require prolonged interactions with high-affinity ligands to integrate sufficient signals for productive T cell activation (80, 81), but do not significantly contribute to activation of T cells by low-affinity ligands such as agonist pMHC, which remain bound to the same TCR on the order of seconds (82).

Triggering of T cell responses by soluble multivalent reagents such as antibodies or pMHC tetramers is often attributed to artificial clustering of the TCR. However, TCRs are already clustered on naïve T cells even in the absence of stimulatory antigens (45, 83). This calls into question the assumption that clustering is a cause rather than a consequence of triggering. An alternative explanation for T cell activation by antibodies and soluble pMHC was proposed by Minguet and Schamel (84); multivalent binding of antibodies or pMHC tetramers can induce changes in the orientation of pMHC molecules in the plasma membrane due to the limited distances between antibody or pMHC binding sites, thereby providing weak mechanical forces on the engaged TCRs. Of note, it has been demonstrated that soluble pMHC dimers linked *via* a short spacer can induce strong T cell activation, whereas soluble pMHC dimers linked *via* long spacers cannot activate T cells (85), consistent with the “permissive geometry” model in which the TCR orientation in the membrane is altered by the smaller pMHC dimers (83). We expressed similar surface densities of 2C11, BC3, and OKT3 scFv on cells, yet elongated 2C11 scFv cannot activate T cells. If artificial clustering is sufficient for TCR triggering, we would anticipate that even elongated 2C11 scFv could activate T cells, but this was not observed.

Studies on chimeric antigen receptors (CARs) (86), bispecific T cell engager antibodies (BiTES) (87), and TCR-dependent bispecific antibodies (88) have reported that the targeted epitope needs to be close to the membrane for optimal triggering. For example, a CAR that bound to a membrane distal epitope of CD22 produced reduced target cell lysis as compared to a CAR that bound to a membrane-proximal position of CD22 (86). The affinity of the scFv used to construct the CAR [*K*_d_ = 8.5 × 10^−8^ M (89)] is similar to the lower affinity 2C11 anti-CD3 scFv used in our study [*K*_d_ = 7 × 10^−8^ M (25, 26, 90)]. Thus, our data showing loss of T cell activation by elongated 2C11 scFv are consistent with the poor target cell lysis by CAR T cells that bound to a membrane distal epitope of CD22. Our data are also consistent with a study in which a BiTE that bound to a membrane-proximal epitope of melanoma chondroitin sulfate proteoglycan more effectively triggered T cell activation and lysis of antigen-positive target cells as compared to a BiTE that bound a membrane distal epitope of the same antigen (87). The anti-CD3 scFv portion of the BiTEs displayed an affinity of ~ 1 × 10^−7^ M (87), which is lower than the affinity the 2C11 scFv used in our study, consistent with a strong dimension-dependent effect on T cell activation of the BiTES, similar to what we observed for membrane-tethered 2C11 scFv. In another study, Li and colleagues found about a 20-fold difference in killing of target cells between bispecific antibodies that bind to the membrane proximal or membrane distal regions of FcRH5. Their bispecific antibody used a humanized mouse antibody (UCHT1v9) with a reported affinity of 4.7 × 10^−9^ M, which is similar to the reported affinity of the intermediate affinity BC3 antibody used in our study [*K*_d_ ~ 8 × 10^−9^ M (27, 28)]. In analogy with membrane-tethered BC3 scFv, a partial but not complete attenuation of T cell activation by the UCHT1v9 bispecific antibody was observed when binding to the membrane distant portion of FcRH5. Comparison of the results of our study and previous studies on bispecific antibodies and CAR T cells might also be influenced by differences in the ligands and readouts. For example, the potency of BiTES and bispecific antibodies may also depend on the affinity of the tumor-binding portion of the molecules (91). T cell killing by perforin/granzyme B might also be less effective when T cells bind to membrane distal regions of receptors. However, even with these caveats, our results appear consistent with previous studies examining the effect of epitope binding position on potency. Our results further suggest that high affinity anti-CD3 antibodies, such as OKT3 [*K*_d_ ~ 5 × 10^−10^ M (28, 29)] may be beneficial for CAR T cells or BiTES that bind to membrane distal epitopes of large surface antigens. Indeed, a CAR T cell directed against a large surface receptor (receptor tyrosine kinase-like orphan receptor 1) displayed enhanced cytokine secretion and killing activity when the affinity of the scFv was increased by 50-fold from a CAR with *K*_d_ ~ 6.5 × 10^−8^ M (92, 93).

In summary, we demonstrate that in contrast to low-affinity ligands, elongation of the tethers of membrane-anchored TCR ligands possessing moderate affinity does not completely abrogate TCR triggering while elongated high-affinity ligands can effectively activate T cells. We propose an *ad hoc* model that we feel well describes our results as well as previous studies on TCR triggering. We hope that our results stimulate more studies to further understand the relationship between ligand affinity, tether dimensions, mechanical force, CD45 segregation, and T cell activation.

## Ethics Statement

Animals were maintained under specific pathogen-free conditions. All animal experiments were carried out in accordance with the recommendations of Institutional Animal Care and Utilization Committee (IACUC) guidelines at Academia Sinica. The protocol was approved by the IACUC and Laboratory Animal Facility and Pathology Core Committee of the Institute of Biomedical Sciences, Academia Sinica. Human whole blood, obtained from healthy donors by the Taipei City Blood Bank, was used under procedures approved by the Academia Sinica Human Subject Research Ethics Committee (protocol AS-IRB01-11069).

## Author Contributions

SRR designed experiments; BMC, MAA, Chien-HsinL, TCC, YCS, YCL, SEC, CCC, THC, and YCL performed experiments; Chau-HwangL provided technical help; and MAA and SRR wrote the manuscript. BMC and MAA contributed equally to this work.

## Conflict of Interest Statement

The authors declare that the research was conducted in the absence of any commercial or financial relationships that could be construed as a potential conflict of interest.
