# Supplementary figures and images for: The Affinity of Elongated Membrane-Tethered Ligands Determines Potency of T Cell Receptor Triggering
Source: Front Immunol. 2017 Jul 10;8:793. doi: 10.3389/fimmu.2017.00793 (PMC5502409; doi:10.3389/fimmu.2017.00793)

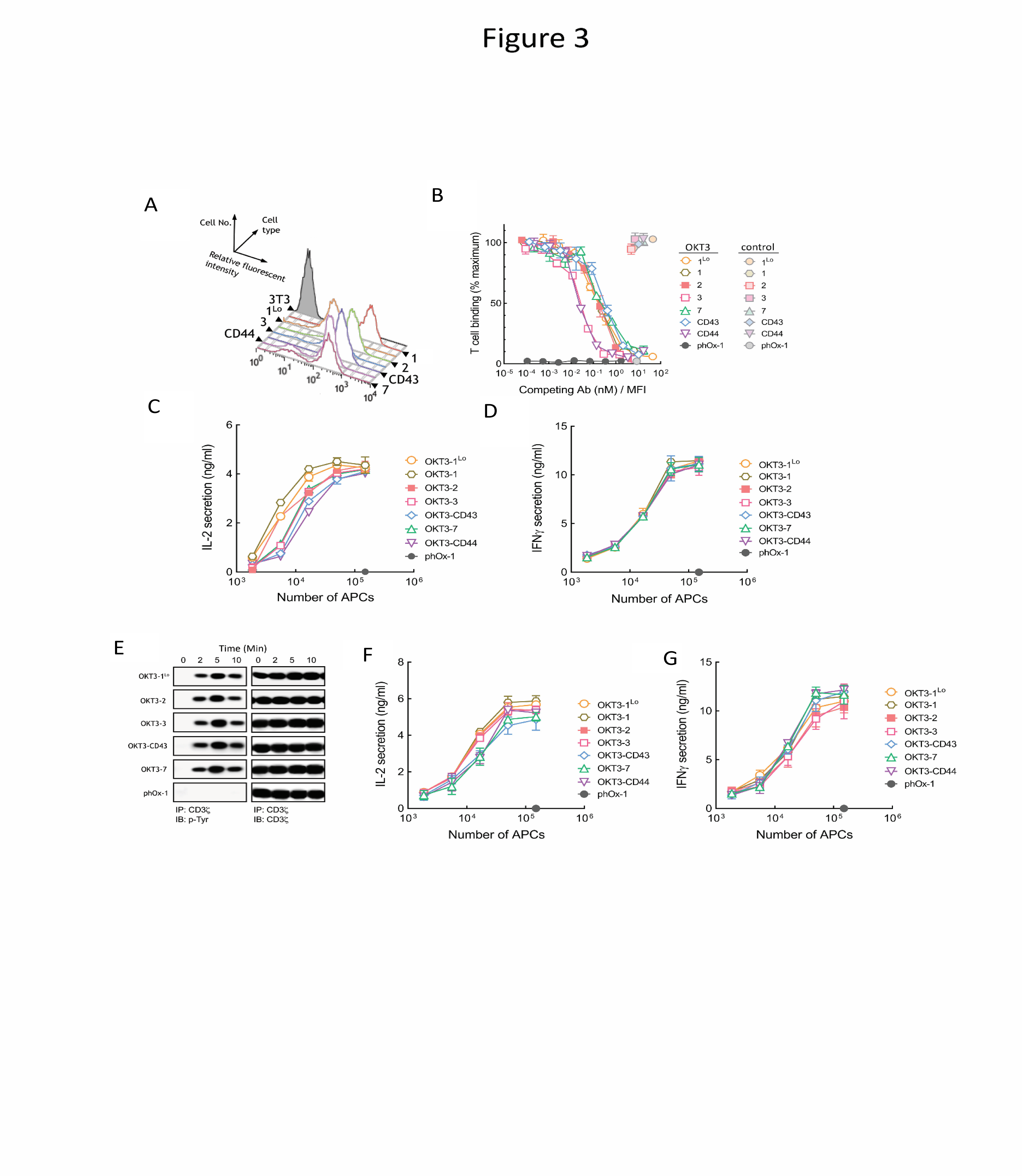

Supplement: Supplementary file 1 [file Figure_3.tif]

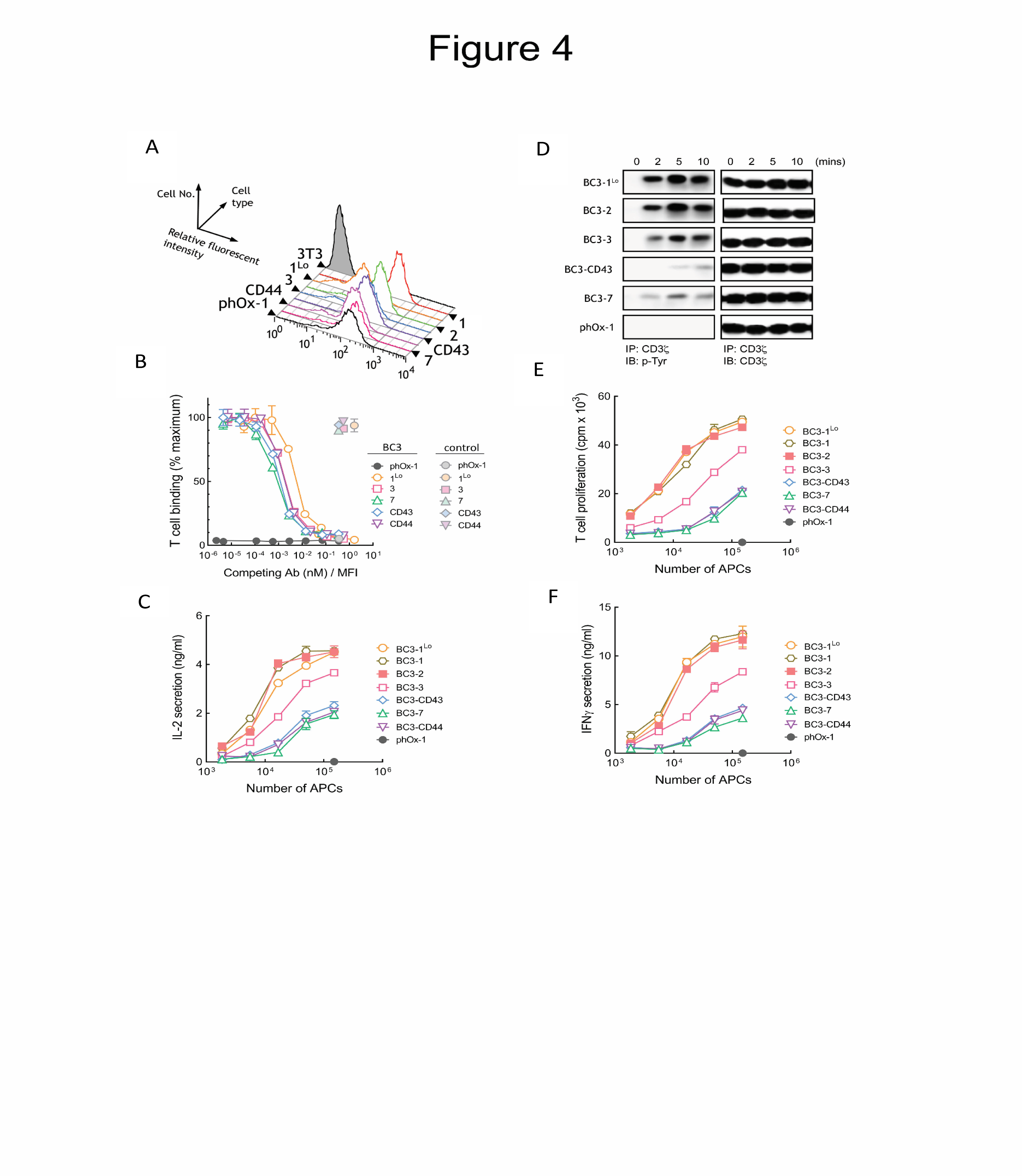

Supplement: Supplementary file 2 [file Figure_4.tif]

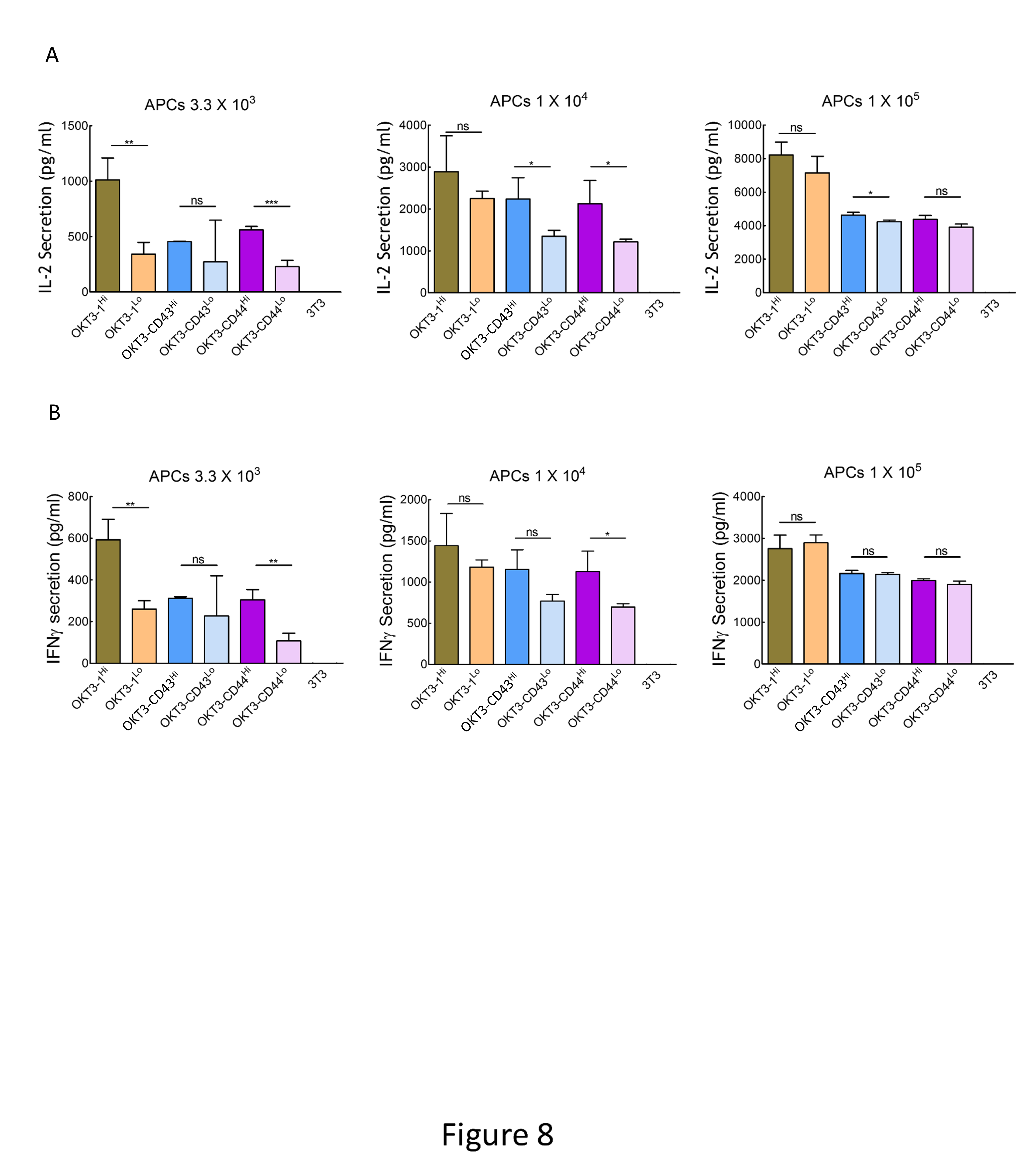

Supplement: Supplementary file 3 [file Figure_8.tif]
