# Supplementary material for: The Affinity of Elongated Membrane-Tethered Ligands Determines Potency of T Cell Receptor Triggering
Source: Front Immunol. 2017 Jul 10;8:793. doi: 10.3389/fimmu.2017.00793 (PMC5502409; doi:10.3389/fimmu.2017.00793)
Supplement: Supplementary file 4 [file Data_Sheet_1.docx]

**The affinity of elongated membrane-tethered ligands determines potency of T cell receptor triggering**

**Bing-Mae Chen, Mohammad Ameen Al-Aghbar, Chien-Hsin Lee, Tien-Ching Chang, Yu-Cheng Su, Ya-Chen Li, Shih-En Chang, Chin-Chuan Chen, Tsai-Hua Chung, Yuan-Chun Liao, Chau-Hwang Lee and Steve R. Roffler**

**Supplementary Materials**

*Supplementary Figures*

*
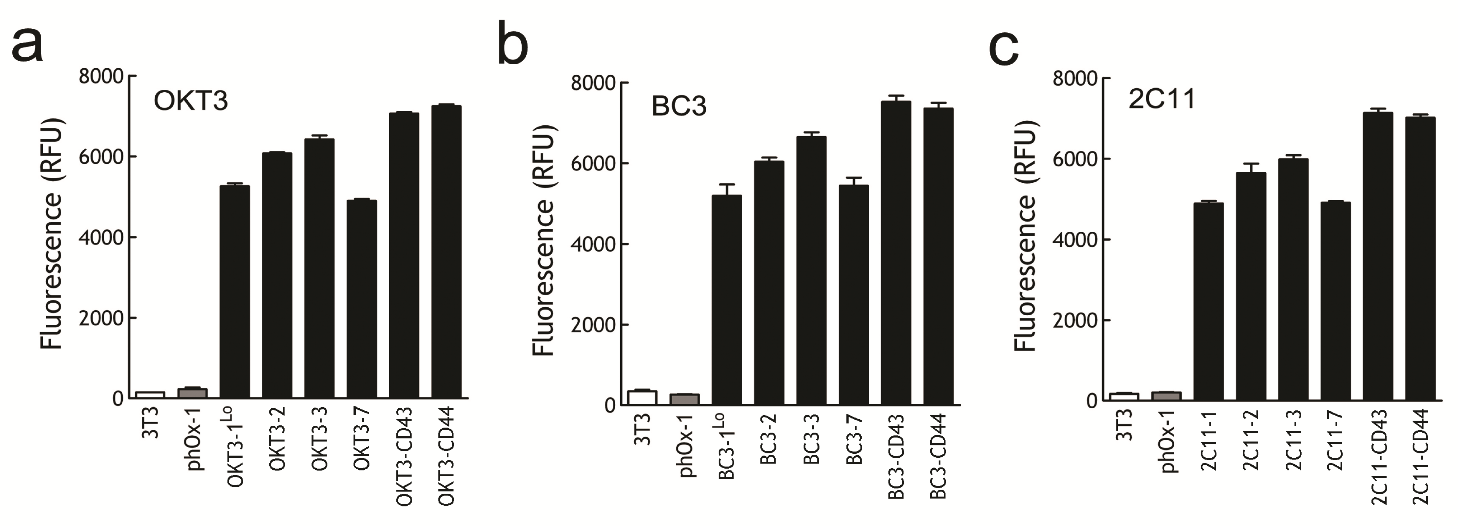
*

**Figure S1. Binding of calcein-labeled T cells to 3T3 cells expressing scFv as measured by fluorescence intensity after washing unbound cells.** **a,** Jurkat T cells binging to 3T3 expressing OKT3 scFv. **b,** Jurkat T cells binding to 3T3 cells expressing BC3 scFv. **c,** 2B4 T cell binding to 3T3 expressing 2C11 scFv. Non-transfected 3T3 or 3T3 cells expressing phOx-1 were used as control.

**
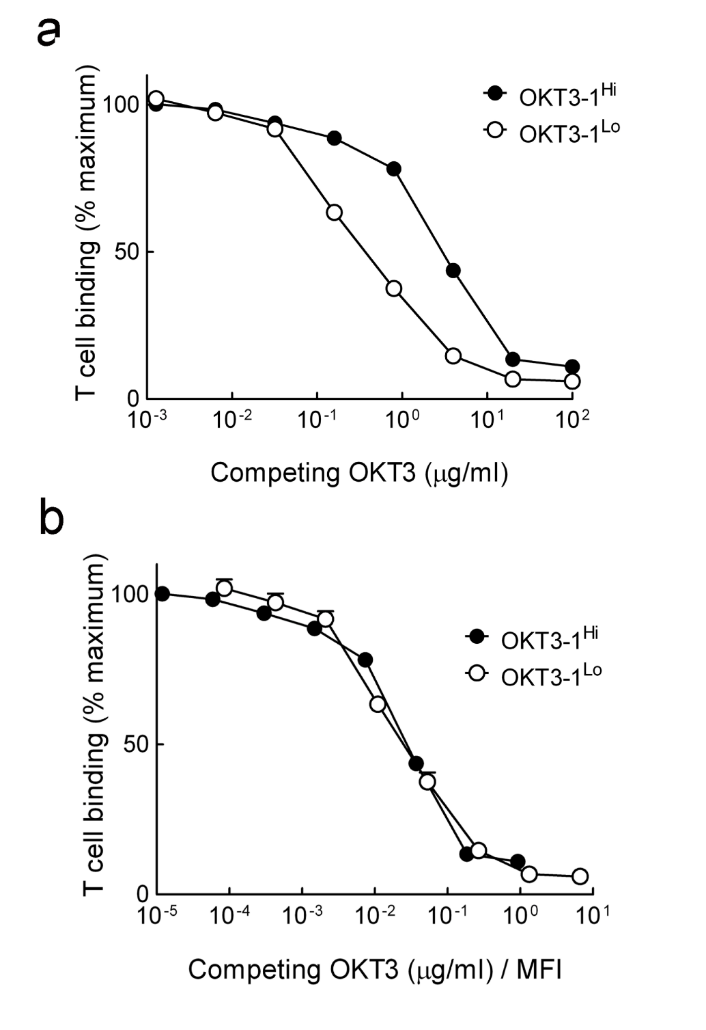
**

**Figure S2. Binding competition by OKT3. a,** Jurkat T cells bound to 3T3 cells expressing high level of OKT3-1 (OKT3-1^Hi^) or low level of OKT3-1(OKT3-1^Lo^) as measured by percentage of mean fluorescent intensity. **b,** the percentage of the mean fluorescent intensities were normalized by the 3T3 scFv expression level **(Fig. 3a).**

**
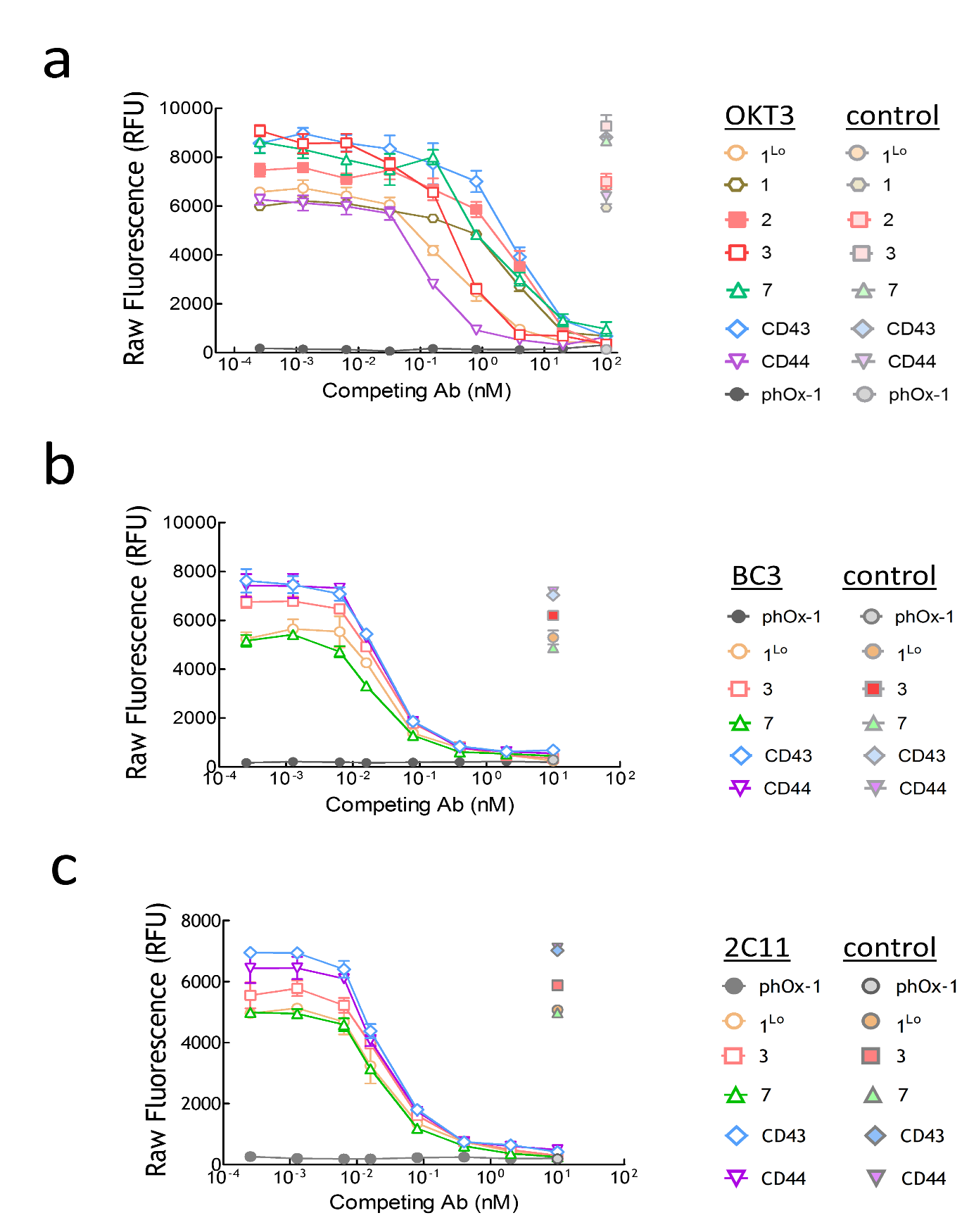
**

**Figure S3. T cell binding to 3T3 APCs.** Calcein-AM-labeled Jurkat T cells (**a**, **b**) or 2B4 T cells (**c**) were incubated with monolayers of 3T3 cells expressing membrane-tethered OKT3, BC3, or 2C11 scFv, respectively. Serial dilutions of competing soluble OKT3 IgG (**a**), BC3 IgG (**b**), or 2C11 IgG (**c**) were included prior to adding T cells. Unbound T cells were washed away and cellular fluorescence of the remaining bound cells was measured (n=3). The y-axis shows the raw fluorescence (RFU) readout of bound T cells whereas the x-axis represents the concentration of added antibodies without normalization to expression level on APCs. Bars, SD.

**
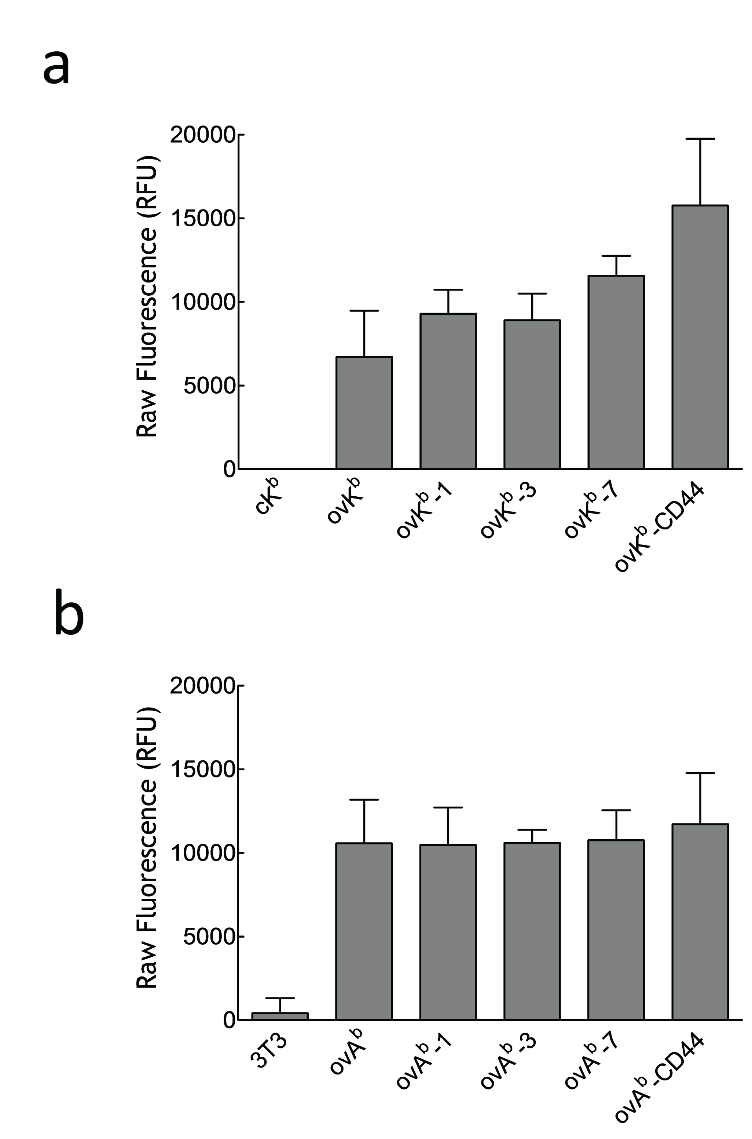
**

**Figure S4. T cell binding to 3T3 APCs expressing membrane-tethered pMHC molecules.** Total T cells purified from splenocytes of OT-I (**a**) or OT-II (**b**) mice were stained with Calceien-AM and added to a monolayer of 3T3 APCs expressing membrane-tethered ovK^b^ (**a**) or ovA^b^ (**b**). Unbound cells were washed out, and the raw fluorescence (RFU) of the remaining bound T cells was measured. Bars, SD, (n=4).

**
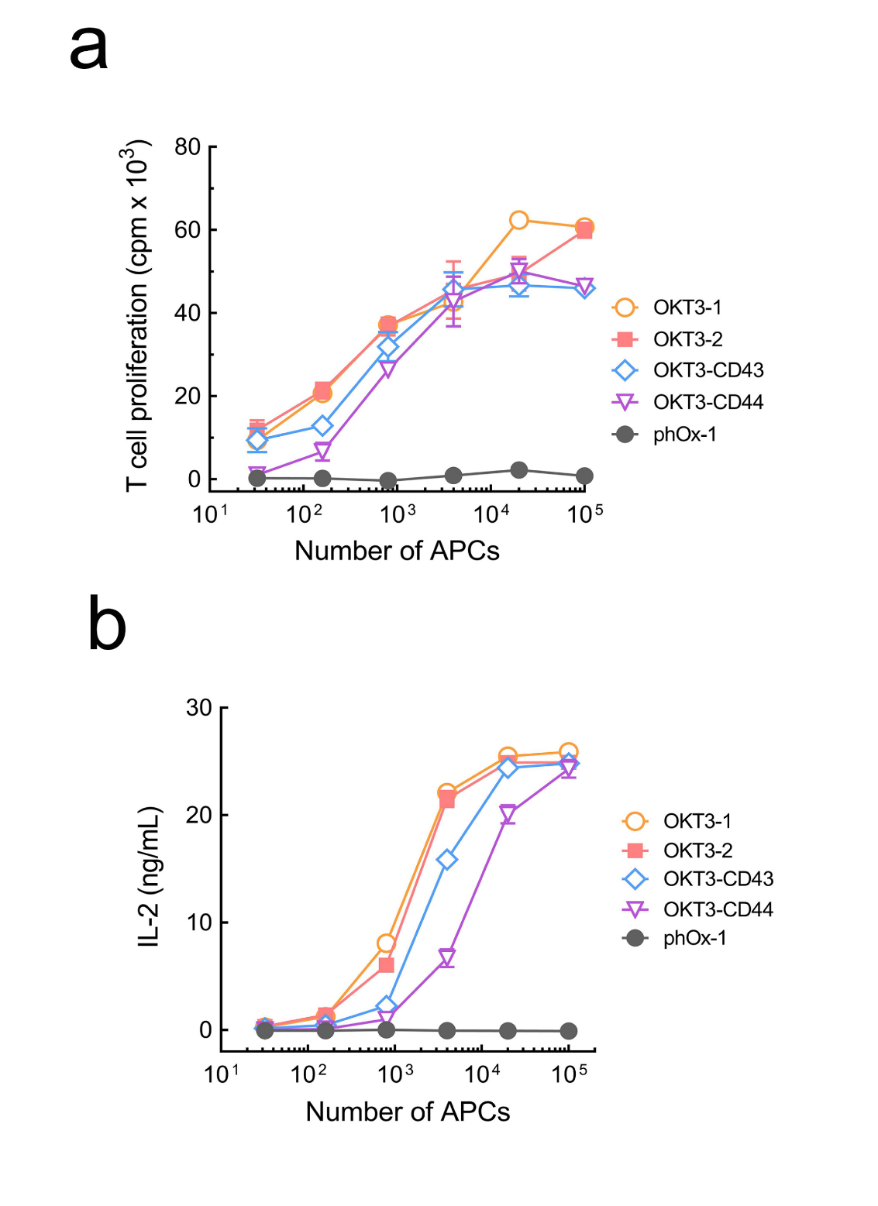
**

**Figure S5. T cell activation by membrane-tethered OKT3 scFv.** The proliferation (**a**) and IL-2 secretion (**b**) of 10^5^ human peripheral T cells incubated for 48 h with the indicated numbers of membrane-tethered OKT3 or control phOx-1 APCs as measured by the incorporation of ^3^H-thymidine into cellular DNA of the T cells. (n=3), Bars, SD.

**
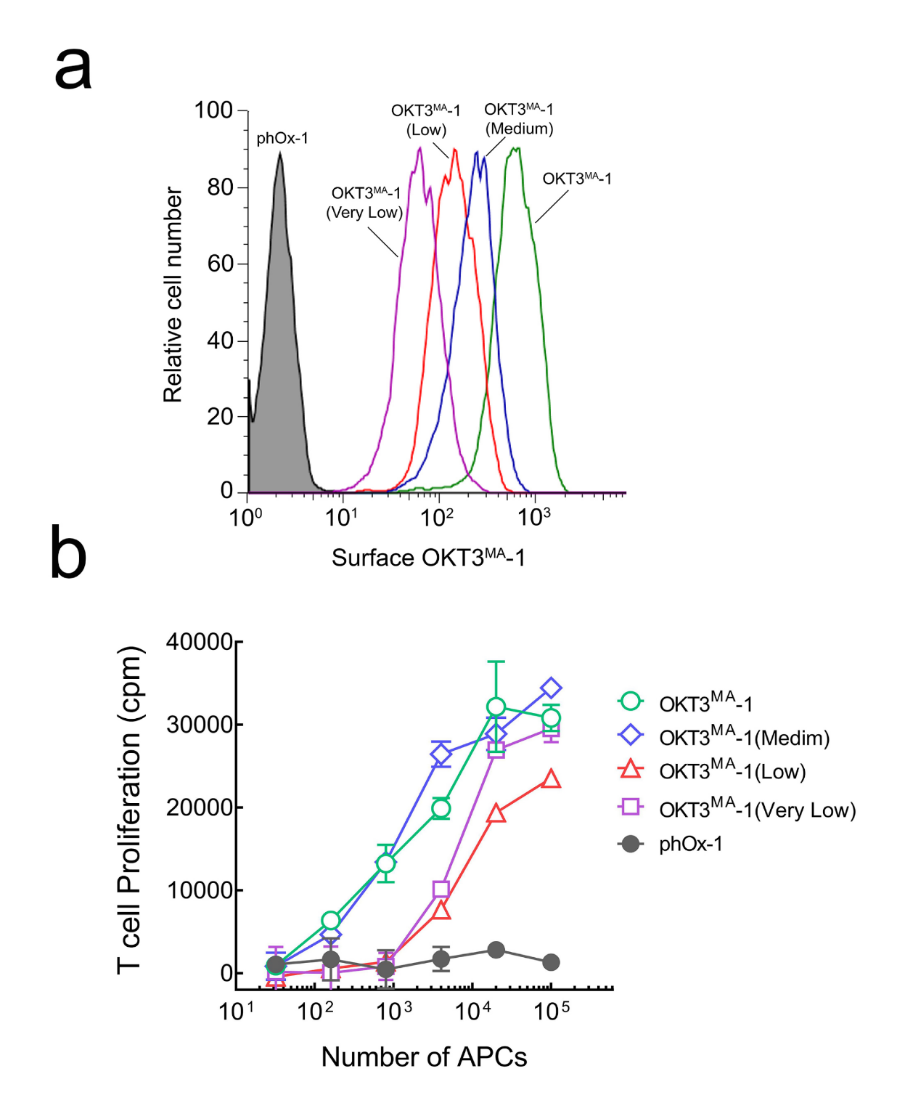
**

**Figure S6. T cell proliferation by APCs expressing different levels of membrane-anchored OKT3^MA^-1 scFv.** **a**, APCs expressing different levels of membrane-tethered OKT3^MA^-1 were analyzed by FACs. **b**, Incorporation of ^3^H-thymidine into cellular DNA of 10^5^ human peripheral T cells incubated with 10^4^ APCs expressing different levels of membrane-tethered OKT3^MA^-1. (n=3), Bars, SD.


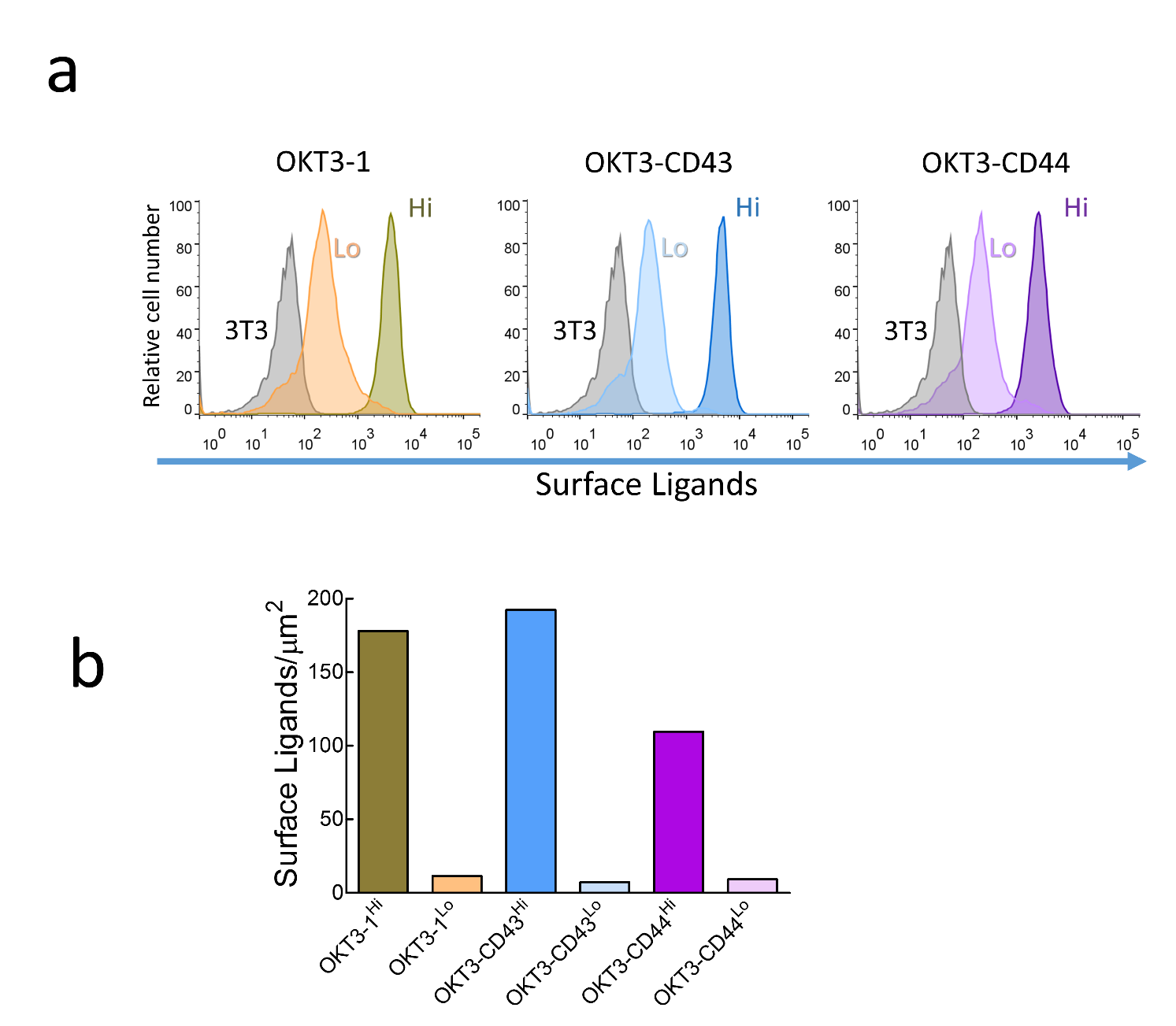


**Figure S7. High and low expression of OKT3 scFv on APCs**. **a**, APCs expressing OKT3 elongated by one Ig-like domain tether (OKT3-1), CD43 (OKT3-CD43) or CD44 (OKT3-CD44), were subjected to two round of sorting to isolate cells expressing low (^Lo^) and high (^Hi^) surface densities of OKT3. The mean density of OKT3 ligands on the surface of 3T3 cells was determined using standard beads in FACS by measuring HA-tag surface expression and secondary FITC-labeled antibody signal. **b**, The surface density of OKT3 was calculated by dividing the antibody binding capacity data by the mean surface area of 3T3 cells.

**
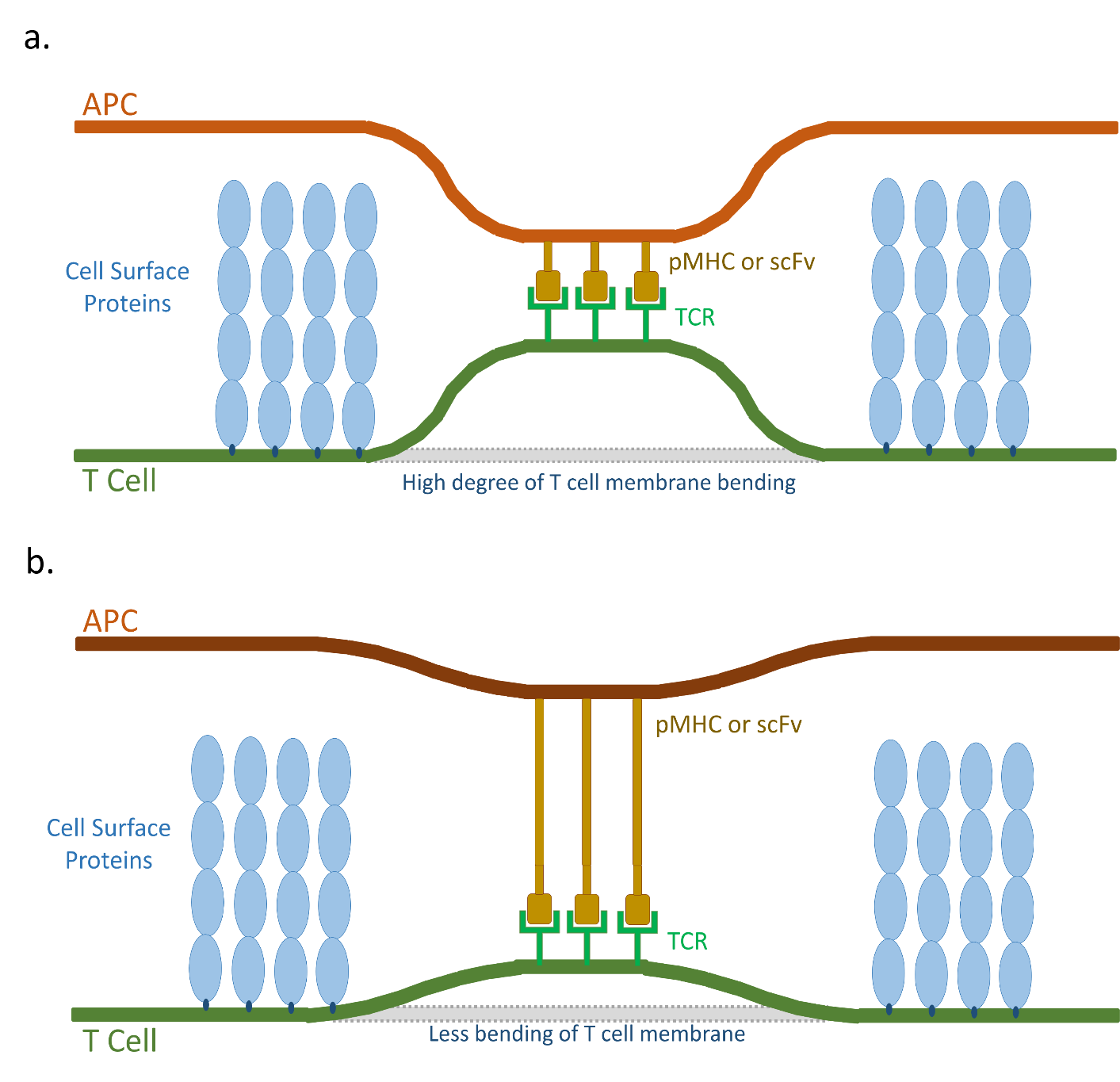
**

**Figure S8. Tensive forces may contribute to TCR triggering.** Different degrees of tensive forces may be generated to facilitate engagement of TCRs. **a,** For short ligands (either scFv or pMHC) to contact and bind TCRs, the T cell and APC membranes are subjected to a high degree of bending to overcome the intercellular repulsion from neighboring large membrane proteins surrounding pMHC and TCR molecules. **b,** Elongation of TCR ligands reduces the amount of membrane bending required for productive engagement of ligand and TCR molecules.

**
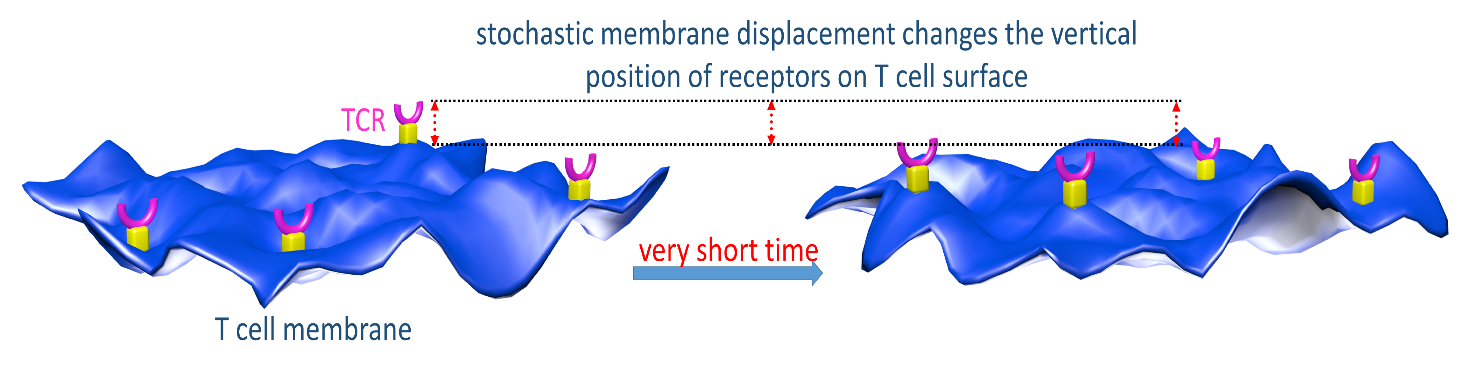
**

**Figure S9. Thermal stochastic membrane fluctuations are conceptualized to contribute to TCR triggering.** T cell and APC plasma membranes display thermally-induced stochastic displacements with frequencies of tens to hundreds of microseconds. The discordant spatial fluctuations of membrane receptors (pMHC and TCRs) in the z-plane may generate periodic forces on engaged TCRs.

*Supplementary Table*

| Tether | Distance (nm) | OKT3 | BC3 | 2C11 | OKT3^MA^ | OKT3^LT^ |
| --- | --- | --- | --- | --- | --- | --- |
| 1 | 10.3 | ++++ | ++++ | ++++ | +++ | + |
| 2 | 12.4 | ++++ | ++++ | ++ | ++++ | ++++ |
| 3 | 14.2 | ++++ | +++ | + | ND | ND |
| CD43 | 18.9 | ++++ | ++ | - | - | - |
| 7 | 24.3 | ++++ | ++ | - | ND | ND |
| CD44 | 40.9 | ++++ | ++ | - | - | - |

**Table S1. S**ummary of TCR triggering based on the normalized IL-2 secretion data in comparison to the spatial distance between APC and T cell in nm (**Fig. 2c**). 4+ means the triggering is close to 100%, 3+ close to 75%, 2+ 30-50%, 1+ less than 25%, - no triggering, and ND means not determined.
